# Amitriptyline-Mediated Cognitive Enhancement in Aged 3×Tg Alzheimer's Disease Mice Is Associated with Neurogenesis and Neurotrophic Activity

**DOI:** 10.1371/journal.pone.0021660

**Published:** 2011-06-27

**Authors:** Wayne Chadwick, Nick Mitchell, Jenna Caroll, Yu Zhou, Sung-Soo Park, Liyun Wang, Kevin G. Becker, Yongqing Zhang, Elin Lehrmann, William H. Wood, Bronwen Martin, Stuart Maudsley

**Affiliations:** 1 Receptor Pharmacology Unit, National Institute on Aging, National Institutes of Health, Baltimore, Maryland, United States of America; 2 Laboratory of Neurosciences, National Institute on Aging, National Institutes of Health, Baltimore, Maryland, United States of America; 3 Center for Neurodegenerative Disease Research, University of Pennsylvania, Philadelphia, Pennsylvania, United States of America; 4 Genomics Unit, Research Resources Branch, National Institute on Aging, National Institutes of Health, Baltimore, Maryland, United States of America; 5 Metabolism Unit, National Institute on Aging, National Institutes of Health, Baltimore, Maryland, United States of America; Federal University of Rio de Janeiro, Brazil

## Abstract

Approximately 35 million people worldwide suffer from Alzheimer's disease (AD). Existing therapeutics, while moderately effective, are currently unable to stem the widespread rise in AD prevalence. AD is associated with an increase in amyloid beta (Aβ) oligomers and hyperphosphorylated tau, along with cognitive impairment and neurodegeneration. Several antidepressants have shown promise in improving cognition and alleviating oxidative stress in AD but have failed as long-term therapeutics. In this study, amitriptyline, an FDA-approved tricyclic antidepressant, was administered orally to aged and cognitively impaired transgenic AD mice (3×TgAD). After amitriptyline treatment, cognitive behavior testing demonstrated that there was a significant improvement in both long- and short-term memory retention. Amitriptyline treatment also caused a significant potentiation of non-toxic Aβ monomer with a concomitant decrease in cytotoxic dimer Aβ load, compared to vehicle-treated 3×TgAD controls. In addition, amitriptyline administration caused a significant increase in dentate gyrus neurogenesis as well as increases in expression of neurosynaptic marker proteins. Amitriptyline treatment resulted in increases in hippocampal brain-derived neurotrophic factor protein as well as increased tyrosine phosphorylation of its cognate receptor (TrkB). These results indicate that amitriptyline has significant beneficial actions in aged and damaged AD brains and that it shows promise as a tolerable novel therapeutic for the treatment of AD.

## Introduction

Alzheimer's disease (AD) is the most common form of dementia associated with memory and cognitive decline. The public health impact of AD amplifies as the proportion of elderly people in the population increases. Classical biochemical hallmarks of AD include the accumulation of amyloid beta (Aβ) peptide oligomers and soluble hyperphosphorylated tau aggregates. The generation of these aggregates has been reported to result in oxidative and inflammatory damage, leading to metabolic failure and synaptic dysfunction. Approximately 40% of AD patients develop depressive symptoms, also known as AD-associated affective disorder, which itself contributes to cognitive decline [1]. To date, the most effective AD therapeutics are cholinesterase inhibitors which can partially improve cognition. However, AD patients suffering from depression, receiving anticholinesterase therapy, display a significant decline in cognitive function compared to depressed AD patients receiving a combination of both anticholinesterases and selective serotonin reuptake inhibitors (SSRI) [2]. Although combinational therapy has shown promise, SSRIs and tricyclic antidepressants (TCAs) have both also been shown to independently reduce the severity of cognitive decline in non-depressed AD patients [3]. Interestingly, it has also been shown that depressed patients receiving a low dose of paroxetine (an SSRI) exhibited a 12% reduction in serum BDNF levels, whereas depressed patients receiving a low dose of the tricyclic antidepressant amitriptyline (AMI) showed a 13% increase in serum BDNF levels [4]. Although TCAs have largely been replaced by SSRIs for the treatment of depression, TCAs such AMI, are currently prescribed for the treatment of neuropathic pain at doses well below antidepressant values, suggesting dose-dependent pluripotent actions of this agent. Interestingly, it was recently shown that AMI possesses neurotrophic activity and can bind to and activate the neurotrophin tyrosine kinase receptor B (TrkB; [5], [6]). Neurotrophins are critical for the maintenance of the peripheral and central nervous systems, and the beneficial effects of neurotrophic factors on neuronal function and AD pathophysiology are well known [7], [8]. An important consideration for the development of successful AD therapeutics is whether the agent retains functional efficacy in aged individuals with advanced pathology and symptomology. To investigate the potential for a therapeutic that possesses efficacy in aged and damaged brains we administered a low, chronic dose of AMI to the triple-transgenic mouse model of AD (3×TgAD; [9]). These 3×TgAD mice exhibit age-dependent Aβ deposition and tau pathology in the hippocampus, which is associated with deficits in spatial learning tasks [9]. We used aged 3×TgAD mice (14 months old), which demonstrated significant AD pathology and cognitive deficits, to determine the potential beneficial effects of AMI upon cognitive function and extant AD pathology. We have previously demonstrated that there are considerable neurophysiological differences between the 3×TgAD mice and their wild-type controls [10], which may differentially affect drug responses. Therefore, in this study we have focused mainly on the direct cognitive effects of AMI treatment on the 3×TgAD mouse model as opposed to any potential effects upon non-pathophysiological animals. The ultimate developmental goal of AD therapeutics is effective prophylaxis, but while early and accurate diagnostic tools are currently unavailable the ability to demonstrate therapeutic efficacy in a cognitively impaired disease-related condition is an important therapeutic step forward.

## Methods

### Animals and treatment

Animal care and experimental procedures followed NIH guidelines and were approved by the National Institute on Aging (NIA) Animal Care and Use Committee under NIA protocol number 293-LNS-2010. Male 3×TgAD mice, 14 months of age, were maintained on a 12 hr light dark cycle in pathogen free conditions. Animals received food and water *ad libitum*. The test group received 100 µg/g body weight amitriptyline-hydrochloride *per os* in their drinking water (Sigma-Aldrich, St. Louis MO) for 4 months (n = 15), and the control group received water (n = 15). Two months into the experiment 50 µg/g BrdU was administered intraperitoneally to both groups daily for 9 days, to examine the potential effects of AMI on neurogenesis. To ensure that any novel AMI-generated BrdU-positive cells were stable and mature, two months were allowed to elapse from initial BrdU injection to the time of tissue harvest. At the end of the study period, animals were euthanized with isoflurane inhalation and decapitation. One half of the brain was carefully dissected on ice, snap frozen and stored at −80°C for further analyses. The other half of each brain was fixed in 4% paraformaldehyde for immunohistochemical analyses.

### Behavioral testing

The Morris water maze (MWM) was used to assess cognitive function. The MWM test protocol has been described in detail previously [11], [12]. Briefly, animals were given four trials per day for seven days to learn the task. On the final day of testing, animals were given a probe trial in which the platform is removed and the amount of time spent in each quadrant by the animal is recorded over a 60 second interval. This probe trial was performed 4 hours, 24 hours and 2 weeks following the final day of training. The probe trial indicates whether the animal can remember where the escape platform was located. In addition to the MWM, we also used the Novel Object Preference (NOP) test to determine learning and memory ability. The animals were placed in opaque boxes (for 15 minutes) which contained two identical objects, and the time spent exploring each object was measured. The following day, one of the objects was replaced with a new object, and again the time spent exploring each object was measured (for 15 minutes). Animals that do not exhibit memory impairment will spend more time exploring the novel object on the second day of testing. Data was reported as the preference index (PI), *i.e.* the total time spent investigating the novel object/total time the animal spent investigating both objects. Subsequently, we measured general activity levels using a standard open field procedure, with 2 zones (I and II), as described previously [11]. The following indices were measured: time spent in specific zone (I or II), total ambulatory counts, vertical activity counts, and total vertical activity time. To assess levels of anxiety, the elevated plus maze was used. The apparatus (San Diego Instruments, San Diego, CA) consisted of two open arms and two closed arms, that extended from a common central platform. The apparatus was elevated to a height of 38 cm above the floor level. Each mouse was placed in the center square facing an open arm and allowed to freely explore the apparatus for 10 minutes. The total time spent exploring the open arms (2 and 4) and the closed arms (arms 1 and 3) was recorded. All behavioral data was collected using Stoelting Any Maze behavioral tracking software (Wood Dale, IL).

### Immunohistochemistry

Hemibrains were fixed in 4% paraformaldehyde (PFA) and stored in 30% sucrose. Fixed hemibrains were sectioned (40 µm) using a vibratome, and then processed using standard protocols [13]. Every eighth section was immunostained using antibodies directed against Aβ (plus antigen retrieval: (5 minutes, 99% formic acid) or hyperphosphorylated tau (AT8, AT180) using ABC Vector Elite and diaminobenzidine kits (Vector Laboratories, Burlingame, CA). All antisera (sources and dilutions) employed are listed in Table S1. Immunohistochemical results were quantified using two different methods by a researcher blinded to the experimental conditions. Firstly, Aβ immunoreactivity was determined by the immunohistochemistry load technique [13]. Load values were determined from selected 420 µm×330 µm fields of immunolabeled sections that were captured and digitized using a video capture system: black and white CCD camera coupled to an Olympus Optical (Tokyo, Japan) BX40 upright microscope. Using NIH Image J (v.1.61), digital grayscale images were converted into binary positive/negative data using a constant threshold limit. Secondly, the numbers of Aβ deposits and AT8-immunoreactive neurons were quantified, as described previously [13].

### Beta- and gamma-secretase assays

Tissue beta- or gamma-secretase enzymatic activity was measured in vehicle-treated or AMI-treated mice using specific fluorometric assay kits (beta-secretase – FP002; gamma secretase – FP003) obtained from R&D Systems (Minneapolis MN, USA). Assays were performed using tissue homogenates (final protein concentration of 1 mg/mL) created using the supplied specific extraction buffer, according to the manufacturers instructions. The assays employ secretase-specific substrate peptides conjugated to the fluorescent reporter molecule EDANS ((5-((2-aminoethyl)amino)naphthalene-1-sulfonic acid) and the molecular quenching agent DABCYL (4-(dimethylaminoazo)benzene-4-carboxylic acid). Cleavage of the substrate peptides relieves the DABCYL-mediated fluorescent quenching of the EDANS moiety, therefore levels of secretase activity (per unit tissue protein) are proportional to the generated fluorescence, measured using a Molecular Devices Spectramax 384-Plus plate reader (Molecular Devices Corporation, Sunnyvale CA).

### Western blotting

Hippocampus and cortex tissue was fractionated using the Qproteome™ Cell Compartment kit, according to the manufacturer's instructions (Qiagen, Valencia CA). All protein extracts were quantified using BCA reagent (ThermoScientific, Rockford IL) before resolution with SDS-PAGE and electrotransfer to PVDF membranes (Perkin Elmer, Waltham MA). Membranes were blocked for western blots, as described previously [10] and primary antibody immune-reactive complexes were identified using alkaline phosphatase-conjugated secondary antisera (Sigma Aldrich) with enzyme-linked chemifluorescence (GE Healthcare, Piscataway NJ) and quantified with a Typhoon 9410 phosphorimager.

### Cell culture and treatment

Dissociated hippocampal and cortical neuron cultures were seeded in poly-D-lysine-coated plates and grown in Neurobasal medium (Invitrogen, Carlsbad CA), and supplemented with B27 (Invitrogen) and 0.5 mM glutamine. Following 7, and in some cases 21, days of maturation, cells were treated with AMI for 3 days, and control cells received phosphate-buffered saline (PBS) vehicle. Cells were then scraped into an NP40-based lysis buffer (250 mM NaCl, 5 mM HEPES, 10% Glycerol, 0.5% NP40, 0.5% Triton-X 100, 2 mM EDTA, protease inhibitors), agitated for 40 minutes at 4°C, and then centrifuged at 14000 rpm/4°C for 15 minutes. Supernatant protein lysates were normalized (1 mg/ml) before western blotting procedures (see above). For acute AMI-stimulation experiments, hippocampal/cortical cells were incubated for 40 minutes in artificial cerebrospinal fluid before AMI exposure (for the time period indicated). After stimulation, cell monolayers were lysed in the NP40-based lysis buffer and prepared for western blotting. Hippocampal and cortical cells for confocal microscopy were seeded in eight well chamber slides (ThermoFisher, Rochester NY). Ten days following seeding, cells were treated with AMI for 3 days. For immunostaining, neurobasal growth media was aspirated and monolayers were washed with PBS and fixed in ice-cold 4% PFA for 20 minutes. Cell monolayers were washed twice with PBS, and then incubated in permeabilization buffer (Tris-buffered saline, 10% goat serum, 0.1% Triton-X 100, 30 minutes). After permeabilization, monolayers were incubated with anti-MAP2 sera. Cells were then incubated with a 1∶2000 dilution of a rhodamine-conjugated anti-mouse antibody for 1 hour at room temperature. Monolayers were washed with PBS between primary and secondary antibody incubations, and also before visualization. For neuronal progenitor cell preparation [14], C57/BL6 embryonic day 14 (E14) pups were employed. Progenitor cells were seeded into poly-L-lysine-coated 6 well plates. Cells were then maintained in DMEM supplemented with B27 supplement (Invitrogen), 25 ng/mL fibroblast growth factor and 25 ng/mL epidermal growth factor. Cells were treated with AMI (10–20 nM), 10 ng/mL brain-derived neurotrophic factor (BDNF) or PBS (vehicle control) for 2 weeks. Cells were then lysed (8% SDS, 125 mM Tris-HCl, pH 7.4) sonicated, and centrifuged (14000 rpm, 10 minutes), before protein concentration determination and normalization (1 mg/mL). All experiments were carried out in triplicate.

### Immunoprecipitation

Primary cortical and hippocampal cells were treated with AMI for the specified time period/dose and subsequently lysed in an ice-cold RIPA buffer. Lysates were agitated for 20 minutes at 4°C before centrifugal clarification (14000 rpm/10 minutes/4°C). Normalized supernatant protein lysates were pre-cleared using protein A/G pre-conjugated agarose beads (EMD Chemicals, Gibbstown NJ). PY20 (anti-phosphotyrosine) pre-conjugated sera (Santa Cruz Biotechnology, Santa Cruz CA) was then added to pre-cleared supernatants with agitation at 4°C. Immunoprecipitates were collected via centrifugation, washed in RIPA buffer and proteins were eluted in 30 µL Laemmli (8% SDS) buffer. Samples were then processed for western blotting. For tissue immunoprecipitations, plasma membrane fractions obtained with Qproteome™ fractionation were normalized, pre-cleared, incubated with anti-PY20 and processed further as described previously [15].

### RNA extraction and microarray analysis

RNA isolation, subsequent cDNA generation, labeling and hybridization to Illumina Sentrix Mouse Ref-8 Expression BeadChips (Illumina, San Diego, CA) was carried out as previously described [15]. Arrays were scanned using an Illumina BeadStation Genetic Analysis Systems scanner and the image data extracted using the Illumina BeadStudio v.3.0. We have deposited the raw transcriptomic data in this manuscript at GEO/ArrayExpress under accession number GSE26836, we can confirm all details are MIAME compliant.

### Bioinformatic analysis

Microarray data were analyzed using DIANE 6.0. Raw microarray data were subjected to filtering and *z* normalization and tested for significant changes, as described previously [15]. Sample quality (in triplicate) was analyzed by principal component analysis and then genes were filtered with a z-ratio cut-off at ±1.5 with an associated false discovery rate <0.3 with a statistical probability (*p*) value≤0.05. These data were further analyzed using ANOVA with significance set at *p*≤0.05. After identifying individual genes that were significantly regulated by AMI treatment, genesets were analyzed using: signaling pathway analysis (pathways defined by the Kyoto Encyclopedia of Genes and Genomes (KEGG) (http://www.genome.jp/kegg/); MSigDB-Parametric Geneset Enrichment analysis (PAGE: (http://www.broadinstitute.org/gsea/msigdb/index.jsp) and scientific abstract latent semantic indexing using GeneIndexer (https://computablegenomix.com/geneindexer). GeneIndexer correlates the strength of association between specific factors (*e.g.* genes or proteins) in a dataset with a user-defined interrogation term [12]. GeneIndexer employs a 2010 murine or human database of over 1×10^6^ scientific abstracts to perform text-gene/protein correlation analysis. LSI facilitates the specific textual interrogation of an input dataset with a specific term, *i.e.* Alzheimer's disease, to ascertain which of the input dataset genes are explicitly associated with the interrogation term. Using LSI algorithms, not only is the direct interrogation term used to analyze the input dataset but also closely correlated additional terms, implicitly associated with the user-defined interrogation term, are also employed in the search patterns. A latent semantic indexing correlation score indicates the strength of association of the interrogation term and the specific genes in the dataset. A highly relevant gene-term correlation yields a large number of explicitly/implicitly associated genes with high LSI correlation scores. Therefore, a strong correlation between the genes in a dataset and a specific user-defined interrogation term yields a large number of correlated genes with high LSI correlation scores.

For the functional clustering of genes into KEGG pathways, using WebGestalt [10], we used a cutoff of ≥2 genes per KEGG pathway at a *p* value of ≤0.05. To assess the degree of KEGG pathways population, a ‘hybrid’ scoring system was used: hybrid score = gene-set enrichment (R)×−log_10_ of the probability (p) of that enrichment. Similar criteria (n≥2 genes per PAGE collection, p≤0.05) were used for the clustering of genes into the MSigDB gene collections. The resultant PAGE z-score is calculated from the individual z-ratios of each experimentally identified gene that populates the specific collection. GeneIndexer genes were only considered to implicitly correlate with the user-defined interrogation term that possessed a latent semantic indexing correlation of ≥0.1.

### Statistical Analysis

For statistical analysis, a non-paired two-way Student's t-test was applied using GraphPad Prism v. 5.0. Statistical significance was considered from p≤0.001 to p≤0.05.

## Results

### AMI alters Aβ and tau deposition

**Figure 1:**
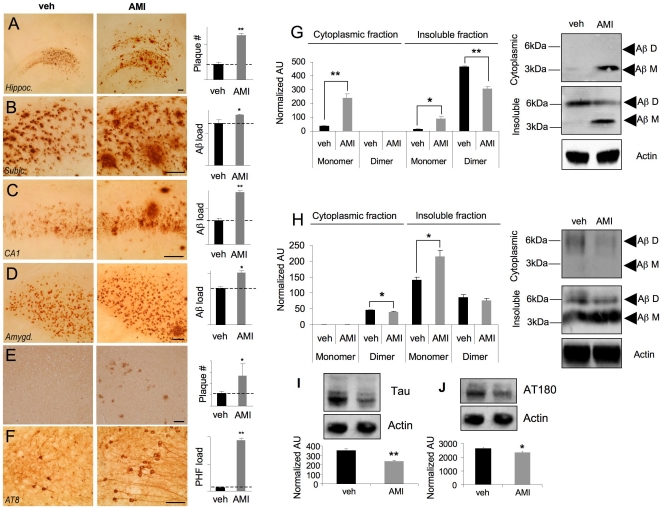
AMI affects Aβ and tau in aged 3×TgAD mice. Vehicle (veh)- and AMI-treated Aβ expression in 3×TgAD hippocampus (A), frontal cortex subiculum (B), CA1 region (C), and amygdala (D). (E) AMI/veh effects on Aβ load in the frontal cortex. (F) AMI/veh effects on hippocampal PHF load. Histograms depict relative quantification of associated panels (A–F). AMI effects on hippocampal (G) or cortical (H) Aβ disposition. (I) AMI/veh effects upon total soluble hippocampal tau levels. (J) AMI/veh effects upon soluble phosphorylated PHF tau. For this and subsequent analyses, values in histograms represent mean ± SEM (n = 6), *p<0.05, ** p<0.01, ***p<0.001.

3×TgAD mice received AMI-hydrochloride for 4 months in their drinking water. Compared to vehicle-treated mice, AMI treatment significantly increased Aβ plaque load in the global hippocampus (Figure 1A:p = 0.0048), subiculum (Figure 1B):p = 0.045, CA1 (Figure 1C:p = 0.001) and amygdala (Figure 1D:p = 0.036). AMI-treatment also significantly increased (p = 0.00098) tau paired helical filaments (PHFs) (Figure 1F), detected using the specific AT8 antisera. We assessed β- or γ-secretase activity in AMI or vehicle-treated animal hippocampus or cortex and detected no significant activity difference in either group (Figure S1). As Aβ plaques, as entities in themselves, may have a variable association with cognitive decline [16], we compared the effect of AMI upon the Aβ dimer to monomer ratio. Using hippocampal subcellular compartmentalization, we found that the AMI-increased Aβ present in the cytoplasmic fraction was of the non-toxic monomeric form, whereas the vehicle-treated group contained almost no monomer (Figure 1G). Both dimeric and monomeric Aβ were noted in the vehicle- and AMI-treated mice insoluble hippocampal compartment. AMI treatment caused a significant increase in monomeric Aβ and a decrease in dimeric Aβ (Figure 1G:p = 0.03). In contrast to hippocampal Aβ expression, only dimeric Aβ was found in the cytoplasmic fraction of cortical extracts (Figure 1H). AMI-treatment significantly reduced cortical cytoplasmic Aβ dimer levels (Figure 1H:p = 0.046). AMI also significantly increased insoluble cortical monomeric Aβ (Figure 1H:p = 0.01). A trend for AMI-mediated decrease in insoluble cortical dimeric Aβ (Figure 1H) was also observed. We also investigated AMI-mediated changes in the microtubule protein tau. PHFs are large complexes of phosphorylated tau [17]. We used standardized ultracentrifugation protocols to investigate AMI effects upon hippocampal tau disposition. Ultracentrifugal supernatants, containing the soluble fraction, were exposed to a ‘total’ tau antibody demonstrating that AMI-treated extracts contained significantly less soluble tau protein (Figure 1I). Using the AT180 anti-tau sera, that recognizes the hyperphosphorylated tau PHF parts of neurofibrillary tangles, we found that AMI-treatment reduced this immunoreactivity in the hippocampus of the 3×TgAD mice (Figure 1J). This suggests that most of the tau present is in the form of PHFs, which are associated with large insoluble inert tangles and are less toxic to the cell than more soluble forms of this complex [18].

### AMI affects expression of multiple neurosynaptic signaling proteins

**Figure 2:**
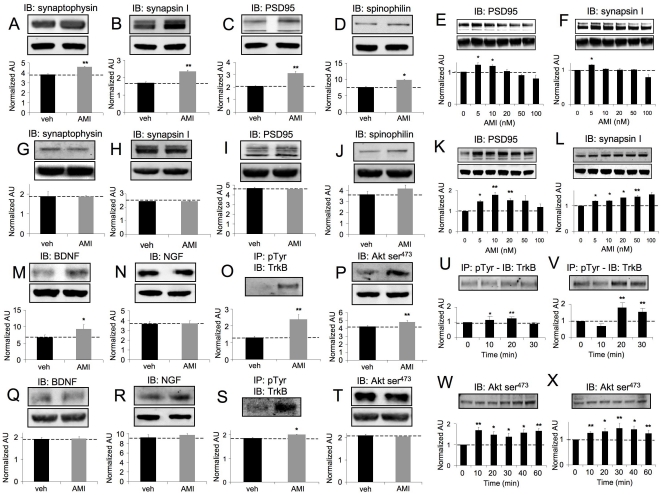
AMI alteration of various synaptic factors. AMI and vehicle (veh)-mediated effects upon hippocampal expression of (A) synaptophysin, (B) synapsin I, (C) PSD95 and (D) spinophilin. AMI effects on PSD95 (E) and synapsin I (F) expression in primary hippocampal cells. AMI/veh-mediated effects upon cortical expression of (G) synaptophysin, (H) synapsin I, (I) PSD95 and (J) spinophilin. AMI effects on PSD95 (K) and synapsin I (L) expression in primary cortical cells. AMI modulation of hippocampal BDNF (M), NGF (M) levels, TrkB tyrosine phosphorylation (O) and Akt-serine (Ser)-473 phosphorylation (P). AMI effects upon TrkB tyrosine phosphorylation in primary hippocampal (U) or cortical neurons (V). AMI regulation of cortical BDNF (Q) or NGF (R) levels, TrkB tyrosine phosphorylation (S) and Akt-1 Ser-473 phosphorylation (T). AMI effects upon Akt-1 Ser-473 phosphorylation in primary hippocampal (W) or cortical neurons (X). Western band intensities were quantified as actin-normalized arbitrary absorbance units (AU).

AMI treatment resulted in significant increases in hippocampal tissue expression of both pre-synaptic (synaptophysin, synapsin I: Figure 2A–B) and post-synaptic (post-synaptic density protein 95 (PSD95), spinophilin: Figure 2C–D) proteins. Acute AMI treatment of isolated murine hippocampal neurons (7 days in culture) also resulted in increased PSD95 and synapsin I (Figure 2E–F) expression. In addition we also demonstrated that similar AMI-mediated effects upon synapsin I and PSD95 expression occurred in primary hippocampal cells after an extended period (21 days) of *in vitro* culture (Figure S2A, B). In murine cortical tissues, minimal changes or small decreases in synaptophysin, synapsin I or PSD-95 (Figure 2G–I) were noted with AMI-treatment, and a modest increase was observed for spinophilin (Figure 2J). Primary murine cortical neurons (7 days in culture) were also responsive to *in vitro* AMI treatment through an increase in synaptic marker proteins (Figure 2K, L). Similar effects of AMI upon cortical PDS95 and synapsin I expression were observed in extended *in vitro* culture (21 days) primary cortical cells (Figure S2C, D). AMI also potentiated hippocampal tissue BDNF, but not NGF levels (Figure 2M,N). AMI significantly increased (p = 0.0036) the phosphotyrosine content of immunoprecipitated hippocampal TrkB (Figure 2O). No changes in cortical/hippocampal TrkA or B receptor levels were seen with AMI treatment (data not shown). AMI also increased hippocampal Akt-1 activity (phospho-Ser473:Figure 2P). AMI did not alter cortical BDNF or NGF expression (Figure 2Q–R). AMI potentiated cortical TrkB phosphotyrosine content (Figure 2S), without elevation of Akt-1 activity (Figure 2T). We assessed the *in vitro* effects of AMI upon these neuroprotective mechanisms with primary murine hippocampal and cortical neurons. Acute AMI stimulation increased TrkB phosphotyrosine content (Figure 2U,V) and activated Akt-1 (Figure 2W,X) in hippocampal and cortical neurons (7 days in culture). These acute effects of AMI stimulation upon TrkB and Akt-1 were also repeated in primary hippocampal and cortical neurons pre-cultured for an extended period as well (21 days: Figure S2 E, F-hippocampus; G, H-cortex). These multiple neurosynaptic effects of AMI indicate an ability to regulate TrkB receptors and stimulate pro-survival/neurotrophic pathways *in vivo* and *in vitro*. Therefore, we subsequently assessed whether AMI could exert pro-cognitive beneficial effects in these aged AD mice.

### AMI significantly improves spatial learning and memory in aged and cognitively-impaired 3×TgAD mice

**Figure 3:**
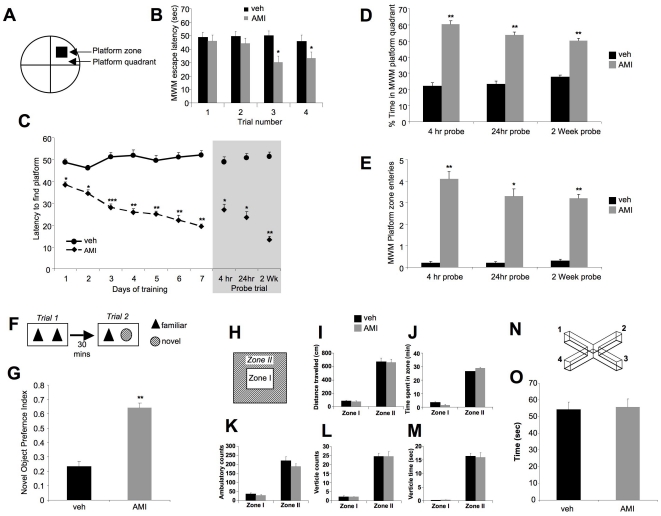
AMI improves learning and memory in 3×TgAD mice. (A) Morris water maze (MWM) used. (B) AMI/veh effects upon day 1 platform escape latency. (C) AMI/veh effects upon MWM platform acquisition measured by escape latency in seconds. Grey panel indicates 3 time points after the seventh day of MWM testing for the probe trial, as described in *Methods*. (D) AMI effects upon time spent in MWM platform quadrant during 3 probe trial time periods after MWM acquisition. (E) AMI effects upon number of platform traverses during the 3 probe trial time periods after MWM acquisition. (F) Novel object preference (NOP) protocol. (G) AMI/veh effects upon 3×TgAD NOP index. (H) Open field test employed. AMI/veh or effects on open field performance: distance traveled (I); time spent in specific zone (J); total ambulatory activity counts (K); vertical activity counts (L) and total vertical activity time (M). (N) Elevated plus maze employed (arms 1 and 3 = dark). (O) AMI effects upon total time spent in open arms 2 and 4.

The effects of AMI treatment upon the cognitive capacity of the aged and cognitively-impaired 3×TgAD was assessed using the standard Morris Water Maze (MWM: Figure 3A). As our treatment is specifically targeted to the amelioration of the specific pathological Alzheimer's cognitive condition, we focused our study on the drug effects in symptomatic transgenic 3×TgAD mice. In addition, as wild-type and 3×TgAD mice demonstrate significant neurosynaptic differences [10], comparison of drug responses between the two may lead to erroneous conclusions regarding drug mechanisms and efficacy. In Figures 3B and 3C it is clear that the control (vehicle-treated) 3×TgAD mice are cognitively impaired as they did not demonstrate a typical reduction in escape latency time across the four platform trials on the first trial day (Figure 3B), or any reduction in time to find the escape platform on subsequent days of MWM training (Figure 3C). AMI-treated mice demonstrated a progressive MWM escape latency reduction over successive trials in their first day of training. The AMI-treated 3×TgAD mice demonstrated a significantly reduced escape latency by the third trial on their first day of MWM training (Figure 3B). Upon repetition of the MWM training over successive days it was clear that AMI treatment greatly enhanced the ability of the 3×TgAD mice to learn the maze task (Figure 3C). Even on the first complete day of MWM testing, AMI-treated mice demonstrated a significantly reduced basal escape latency (averaged across the four trials per day per mouse), compared to vehicle-treated 3×TgAD mice (Figure 3C:p = 0.0197). On each successive trial day the AMI-treated mice demonstrated a progressive reduction in the time to reach the escape platform. Removal of the hidden platform, creating a classical MWM probe trial, demonstrated that AMI-treated mice effectively retained the memory of the platform position after 4 hrs, 24 hrs and even 2 wks post platform testing (Figure 3C, shaded area). The probe-trial search pattern, indicated by time spent in the relevant quadrant zone during the probe trial, of vehicle-treated 3×TgAD mice was random (≤25%), whereas that of AMI-treated 3×TgAD mice was more specific (≥50%: Figure 3D). The number of platform zone entries of AMI-treated 3×TgAD mice during the probe trial was significantly greater than that for the vehicle-treated group at each time point (Figure 3E). We also employed an additional test of memory-related cognitive function, *i.e.* the novel object preference (NOP) test (Figure 3F). AMI-treated animals demonstrated a significantly greater preference for interaction with the novel object than the 3×TgAD vehicle-treated mice (Figure 3G:p = 0.00066). Thus, our findings from the NOP test confirmed our findings from the MWM, in that AMI was able to effectively rescue the cognitive impairment of the aged 3×TgAD mice. It is however known that the MWM test can cause increased anxiety in mice, particularly the 3×TgAD strain [11]. Improved MWM performance therefore may be due to an anxiolytic effect that can be induced by TCAs such as AMI. To investigate whether the AMI-enhanced MWM performance was anxiolytically-based, we performed open field and elevated plus maze testing. No difference in any of the open field (schematically represented in Figure 3H) measurement indices between AMI- or vehicle-treated 3×TgAD mice were noted (Figure 3I–M). Any significant anxiolytic action of AMI would likely affect one or more of these parameters. Additionally, no significant anxiolytic effects in AMI-treated 3×TgAD animals was observed in the elevated plus maze (schematically represented in Figure 3N) test (Figure 3O), again suggesting that pro-cognitive AMI actions may lie outside typical antidepressant/mood stabilization effects, and may be more associated with neurotrophic functions.

### AMI activates genomic transcription patterns related to neurotrophic rather than antidepressant actions

**Figure 4:**
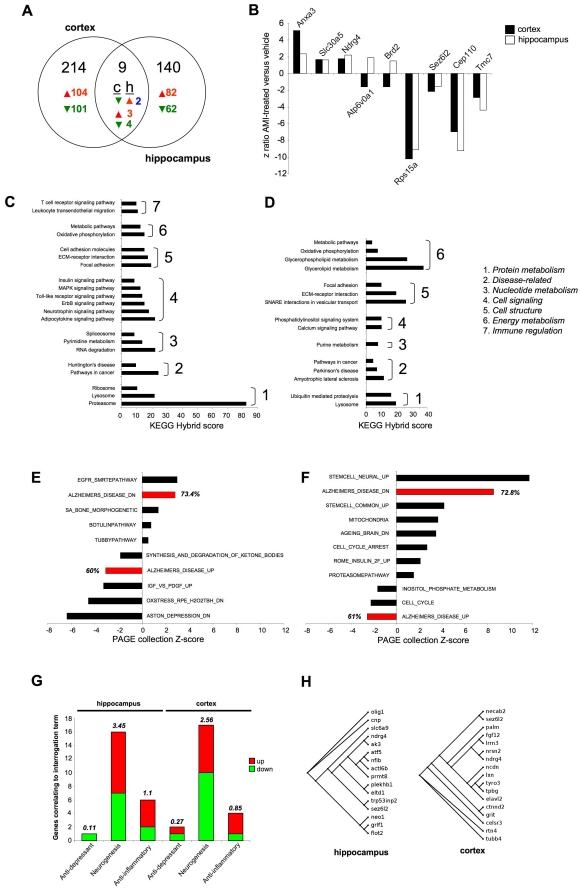
Hippocampal and cortical AMI genotropic actions. (A) Venn analysis of AMI-significantly-regulated transcripts in 3×TgAD cortex (c) and hippocampus (h) (red-upregulation: green-downregulation; blue-diverse regulation). (B) Commonly AMI-regulated genes and z-ratios (cortex-black: hippocampus-white). KEGG signaling pathway population, quantified as a ‘hybrid’ score (see *Methods*) by hippocampus (C) and cortex (D) AMI-regulated genes. (E) MSigDB PAGE collection population, quantified as aggregate z-score, by significant AMI-regulated hippocampal (E) or cortical (F) genesets (PAGE collections directly associated with AD-highlighted in red). Percentage gene population of PAGE collection by the input AMI-regulated geneset is indicated for each red histogram bar. (G) Latent semantic indexing (LSI) GeneIndexer term interrogation of significantly AMI-regulated hippocampal or cortical genes. Numbers of AMI-regulated genes implicitly correlating with the specific interrogation term are depicted by the size of the colored bars (red-upregulated: green-downregulated). Total LSI correlation score of all identified genes is indicated above each histogram bar. (H) Dendrogram association of AMI-regulated hippocampal/cortical neurogenesis-associated genes.

To assess, in an unbiased manner, the potential molecular mechanisms mediating the pro-cognitive actions of AMI we performed transcriptional analysis of treated versus control hippocampal and cortical tissues. AMI-treatment, compared to vehicle control, significantly altered transcription of 223 (Table S2) and 149 (Table S3) genes in the hippocampus and cortex respectively. A small overlap of significantly regulated genes between the two tissues was noted (Figure 4A). Of the common transcripts, 3 were upregulated, annexin A3 (*Anxa3*), solute carrier family 30 (zinc transporter) member 5 (*Slc30a5*) and N-myc downstream regulated gene 4 (*Ndrg4*: Figure 4B). The 4 common downregulated transcripts were: ribosomal protein S15a (*Rps15a*), seizure related 6 homolog like 2 (*Sez6l2*), centrosomal protein 110 kDa (*Cep110*) and transmembrane channel-like gene family 7 (*Tmc7*). Only 2 common transcripts were differentially regulated, ATPase, H^+^ transporting, lysosomal V0 subunit A1 (*Atp6v0a1*) and bromodomain containing 2 (*Brd2*) were downregulated in the cortex and upregulated in the hippocampus by AMI treatment (Figure 4B). To assess the AMI transcriptomic response patterns at an integrated, higher functional level, we subjected the significantly regulated genesets to unbiased computational signaling pathway analysis. AMI-regulated genesets were subjected to pathway enrichment analysis using the KEGG (Kyoto Encyclopedia of Genes and Genomes) database. KEGG database interrogation with the AMI-regulated hippocampal geneset resulted in the significant (p≤0.05) population of signaling pathways linked to cell signaling and neurosynaptic activity (*neurotrophin signaling*, *ErbB signaling*, *Toll-like receptor signaling*, *MAPK signaling*), protein metabolic pathways (*proteasome*, *lysosome*, *ribosome*) and cellular architecture pathways (*focal adhesion*, *ECM-receptor interaction*, *cell adhesion molecules*) (Figure 4C). KEGG database interrogation with the AMI-regulated cortical geneset resulted in the population of a similar cluster of signaling pathways compared to the hippocampus, however fewer cell signaling pathways were populated (6-hippocampus, 2-cortex: Figure 4D). The Molecular Signatures Database (MSigDB) contains interrogatable, curated gene collections that represent complex physiological experimental paradigms, as opposed to the more rigid signaling pathways of the KEGG database. Therefore, interrogation of the MSigDB may also assist analogy of the AMI effects with previously identified physiological paradigms. The significantly populated (p≤0.05) MSigDB geneset collections from the hippocampal (Figure 4E) or cortical (Figure 4F) AMI-regulated transcripts demonstrated a strong pro-cognitive therapeutic profile. In the hippocampus, AMI treatment resulted in the upregulation of a collection of genes shown to be downregulated in AD (*ALZHEIMERS_DISEASE_DN*), with a concomitant downregulation of genes typically upregulated in AD pathology (*ALZHEIMERS_DISEASE_UP*). In both of these gene collections, we identified the significant AMI-regulation of over 60% of the total number of base-set genes in each collection (Figure 4E). Interestingly, AMI was unable to reverse the presentation of genes related to a depressive phenotype (*ASTON_DEPRESSION_DN*). An effective reversal of AD-related pathology genes in cortical tissues was also seen in AMI-regulated MSigDB collections (Figure 4F). We also noted a strong AMI-mediated upregulation of neural stem cell activity (*STEM_CELL_UP*, *STEM_CELL_COMMON_UP*), changes in cell cycle (*CELL_CYCLE_ARREST*, *CELL_CYCLE*) and reversal of age-related genetic changes (*AGEING_BRAIN_DN*).

A potential mechanism by which AMI could enhance cognitive function, in the presence of increased classical AD pathology, could be a neuronal re-population of CNS regions via adult neurogenesis. New neurons could support or re-populate disrupted signaling networks to achieve cognitive enhancement. As we had seen a strong pro-neurotrophic effect in the protein expression, enhanced cognitive function and stem-cell related activity in the geneset analysis (with negligible antidepressant-like activity), we next assessed whether AMI-controlled genesets were more related to neurogenic actions than antidepressant activity. Using a latent semantic indexing (LSI) process (Computable Genomix, GeneIndexer), the correlation to curated scientific abstracts of input dataset genes with a user-defined interrogation term can be assessed. Using GeneIndexer interrogation we noted that considerably more AMI-regulated genes correlated with the ‘*neurogenesis*’ term, compared to the ‘*antidepressant*’ term in both hippocampal and cortical datasets. We also noted a strong geneset correlation to the term ‘*anti-inflammatory*’, a function previously associated with AMI [19]. Sixteen and seventeen AMI-regulated genes implicitly correlated with neurogenesis in the hippocampus and cortex respectively (Figure 4H). Despite a similar number of AMI-regulated neurogenesis-associated genes in each tissue, the accumulated LSI correlation score for all these genes was greater for the hippocampal (3.45) compared to the cortical set (2.56). These unbiased bioinformatic data analyses suggested that AMI could be exerting neurotrophic actions in the aged 3×TgAD mice, which we subsequently investigated.

### AMI promotes *in vitro* neuronal development and hippocampal adult neurogenesis

**Figure 5:**
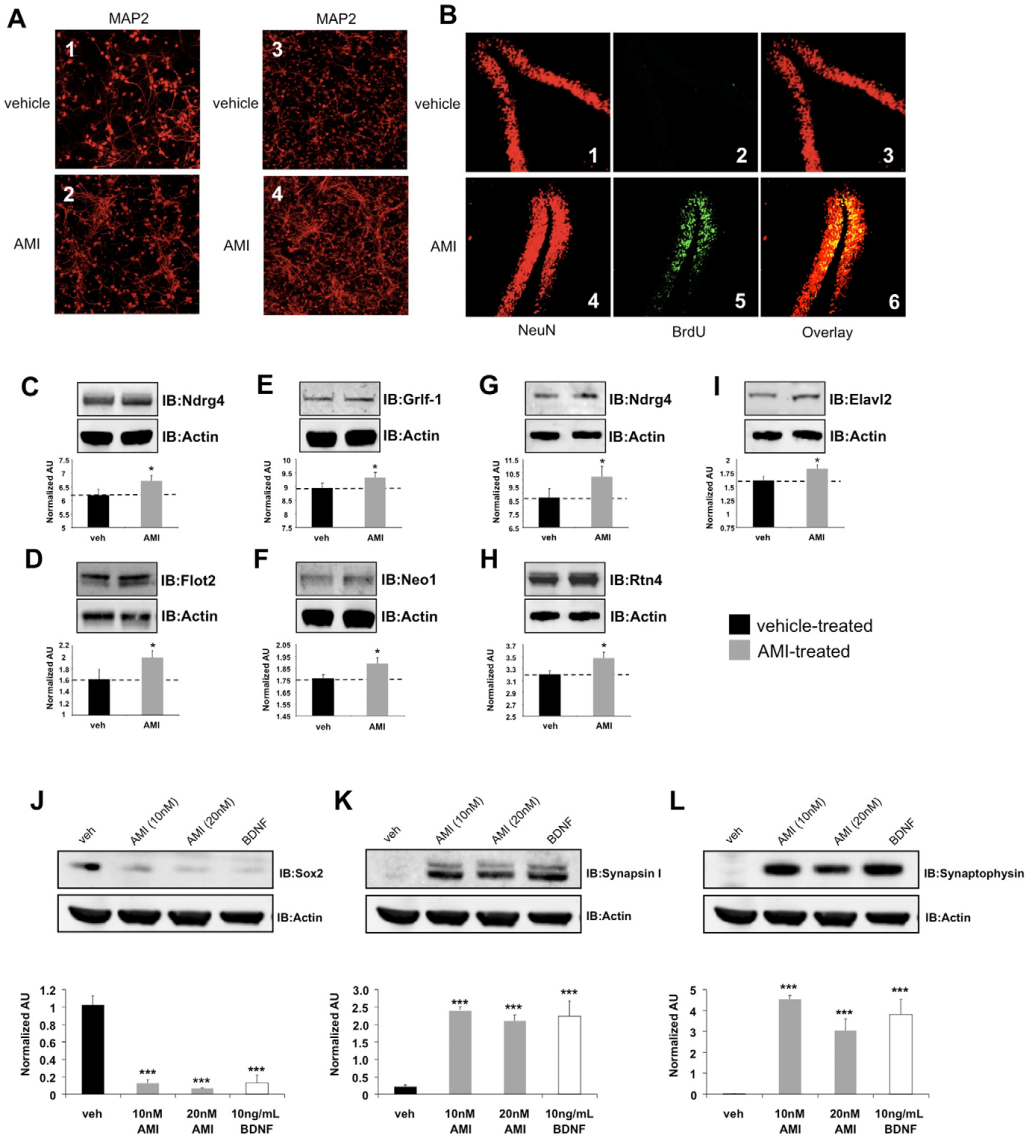
AMI activates neuronal developmental processes. (A) AMI (10 nM, 72 hrs) effect upon primary hippocampal (veh-treated, 1: AMI-treated, 2) and cortical (veh-treated, 3: AMI-treated, 4) cell morphology, indicated with anti-MAP2 immunoreactivity. (B) AMI/veh effects upon adult neurogenesis in hippocampal DG (veh: NeuN-1, BrdU-2, overlay-3; AMI: NeuN-4, BrdU-5, overlay-6). AMI-mediated alterations of hippocampal *Ndrg4* (C), *Flot2* (D), *Grlf1* (E) and *Neo1* (F) expression. AMI-mediated alterations of cortical *Ndrg4* (G), *Rtn4* (H) and *Elavl2* (I) expression. Western band intensities were quantified as actin-normalized arbitrary absorbance units (AU:n = 3 individual experiments per protein assessed). Two week treatment of murine neural progenitor cells with AMI (10–20 nM: grey bars) or BDNF (10 ng/mL: white bars) affects Sox2 (J), synapsin I (K) and synaptophysin (L) expression, compared to vehicle treated (black bars) cells. The associated histograms depict the mean ± SEM western band intensity (normalized AU) data from three independent experiments.

We next directly assessed, *in vitro* and *in vivo*, the potential structural neurotrophic actions of AMI. Application (72 hrs) of the Akt-1/Trk-stimulating AMI doses (10 nM) to primary mouse hippocampal or cortical neurons potentiated neurite formation and axonal growth, compared to vehicle-treated cells (Figure 5A). To assess *in vivo* neurotrophic actions of AMI, bromo-deoxyuridine (BrdU) or a vehicle control was administered intraperitoneally to 3×TgAD mice (control and AMI-treated), daily for 9 days. BrdU stably incorporates itself into DNA of newly dividing cells, acting as a marker of new cell development. 3×TgAD mice can display adult neurogenic behavior, but typically newly developed cells undergo apoptosis and are unable to develop into mature neurons. To ensure that any novel AMI-generated BrdU-positive cells were stable and mature, two months was allowed (with continued maintenance of vehicle or AMI application) to pass from initial BrdU injection to the time of tissue harvest. One of the primary CNS regions of adult neurogenesis is the hippocampus, with the dentate gyrus (DG) being one of the most important sub-regions. AMI treatment profoundly increased the number of BrdU positive cells in the DG, compared to vehicle-treated animals (Figure 5B). Additional hippocampal sub-regions, *i.e.* CA1-CA3 demonstrated only minimal levels of AMI-mediated neurogenesis compared to the DG (data not shown). Nearly all of the BrdU positive cells were co-immunoreactive to NeuN staining, indicating their mature neuronal status. Vehicle-treated animals presented no BrdU positive cells in any of the hippocampal regions. We also detected AMI-mediated elevation of expression of multiple hippocampal/cortical proteins associated with neurogenesis and neuronal growth/development [20]–[23], many of which we also identified using our transcriptomic analysis. AMI induced a consistent potentiation of *Ndrg4* (Figure 5C), flotillin-2 (*Flot2*: Figure 5D), glucocorticoid receptor DNA binding factor 1 (*Grlf1*: Figure 5E) and neogenin-1 (*Neo1*: Figure 5F) hippocampal protein expression. AMI also potentiated cortical expression of *Ndrg4* (Figure 5G), reticulon-4 (*Rtn4*: Figure 5H) and embryonic lethal abnormal vision drosophila homolog-like 2 (*Elavl2*: Figure 5I). It appears therefore that AMI treatment was able to induce the creation of a stable cohort of new neurons in the symptomatic, aged 3×TgAD mice. We therefore next investigated whether AMI was able to directly control the development of cells to a neuronal lineage in an *in vitro* environment. To assess the neuronal developmental capacity of AMI treatment, either AMI or BDNF was applied to isolated murine neural progenitor (NP) cells for two weeks. AMI-treatment was able to significantly reduce expression of Sox2 (sex determining region Y-box 2), a marker of undifferentiated progenitors (Figure 5J), while simultaneously increasing the expression of mature neurosynaptic markers synapsin I (Figure 5K) and synaptophysin (Figure 5L). The effects of AMI closely mirrored those of BDNF upon the NP cells (Figure 5J–L) with respect to Sox2, synapsin I and synaptophysin expression, indicating a strong developmental-neurotrophic functionality to AMI activity.

## Discussion

We have shown that AMI treatment in aged and cognitively-impaired 3×TgAD mice resulted in increases of potentially ‘non-toxic’ Aβ plaque load in both the hippocampus (Figure 1A) and the frontal cortex (Figure 1E), as well as increases in the hippocampal PHF load in 3×TgAD mice (Figure 1F). Despite the seemingly increased classical AD pathology, AMI-treated 3×TgAD mice showed significant increases in neurosynaptic protein expression and neurotrophin receptor/pro-survival kinase activity, enhancement of spatial learning and memory as well as adult hippocampal neurogenesis (Figures 2,3,5). Increased Aβ aggregation was not associated with increases in β- or γ-secretase enzyme activity (Figure S1). A possible explanation for this altered amyloid deposition may be linked to zinc-modulating factors such as the zinc transporter, *Slc30a5*. This transcript was elevated by AMI-treatment in both the hippocampus and cortex (Figure 4B). *Slc30a5*, also known as ZNT5, is associated with neuronal response mechanisms to elevated amyloid plaque load in AD [24]. ZNT5 expression is associated with Aβ plaques and facilitates translocation of cytoplasmic zinc ions into the Golgi apparatus [25]. ZNT5 may therefore participate in the AMI response (*i.e.* elevated plaque load) process in the 3×TgAD mice, by transporting cytoplasmic zinc ions into the Golgi apparatus and incorporating them into newly synthesized metalloproteins. The large Aβ histological plaques seen with AMI treatment may also be part of the therapeutic cognitive effects observed. Large insoluble plaques have been shown to be unreactive and relatively harmless to normal cell functioning [26]. The large Aβ plaques induced by AMI-treatment may also act as molecular sinks for any soluble dimeric Aβ. The majority of the Aβ in AMI-treated mice, in both hippocampal cytosolic and insoluble fractions, consisted of non-toxic monomer and very little toxic dimer (Figure 1G). Control (vehicle-treated) 3×TgAD animals however contained mainly toxic dimeric Aβ and little monomer. Aβ oligomers are considered highly cytotoxic, with dimers being the smallest form of toxic oligomers [26], [27]. The oligomeric assemblies of Aβ transiently arising in the path of fibrillization of several peptides and proteins associated with amyloid disease are typically seen as the primary or even sole cytotoxic species of amyloid [28]–[32]. Evidence also indicates that intermediate amyloid oligomer assemblies demonstrate a broad spectrum of abilities to impair cell physiology and viability, for example, AD-related neuroinflammation is more specifically associated to the presence of larger fibrillar Aβ [33], whereas smaller Aβ oligomers impair neuronal long-term potentiation [28], raise endoplasmic reticulum stress [34] and induce cell death following an aggregation state-specific uptake [35]. It is clear that there is an intricate and complex relationship between Aβ structure and neurophysiological outcomes. Hence, monomers of Aβ may even be neurotrophic and beneficial for neuronal survival [36]. The AMI-increased Aβ monomer levels could therefore constitute part of the observed DG neurogenesis (Figure 5B). In addition to AMI-mediated changes in Aβ, the increased PHF load seen with AMI may also contribute to its therapeutic action. Large insoluble PHFs, like large Aβ plaques, may also be unreactive and relatively benign, whereas soluble PHFs cause cellular dysfunction [18]. We found that AMI significantly reduced soluble and phosphorylated tau compared to the control group (Figure 1I, J). In accordance with these potentially therapeutic effects of AMI upon neurophysiology, AMI also elevated *in vivo* hippocampal TrkB tyrosine phosphorylation and downstream Akt-1 activation levels (Figure 2 O,P). Direct application of AMI to primary hippocampal cells recapitulated this effect, suggesting that AMI may directly activate the TrkB receptor. Recent reports have also suggested that AMI may possess a direct neurotrophin-like action, independent of the cognate ligands for Trk family receptors [6]. We also found that AMI also potentiated hippocampal BDNF tissue levels, suggesting a potential ‘rejuvenation’ of the 3×TgAD hippocampus, as reductions in BDNF have been associated with increasing age [37]. Supporting this posit is our demonstration of AMI-mediated stable DG neurogenesis (Figure 5B) and the *in vitro* ability of AMI to induce NP cell development into neuronal tissue (Figure 5I–K). It is possible that these new hippocampal DG neurons generated by AMI treatment of the 3×TgAD mice could assist in the creation of new neural networks that facilitate the pro-cognitive effects seen in our behavioral tests (Figure 3). Investigation of the alterations in neurite architecture and dendritic spine density may reveal the relative importance of such a process in the future. Newly developed cells may also be more effective at releasing neurotrophic factors, such as BDNF. Reinforcing an AMI neurotrophic action, we found that AMI elevated *Ndrg4* expression in both the hippocampus and cortex (Figures 4B, 5C,G). *Ndrg4* was primarily identified as a gene controlling brain development [38] and has recently been shown to promote neuronal survival, positively regulate neurite outgrowth as well as regulate neurotrophin receptor signaling [20]. AMI also increased *Anxa3* expression in both the hippocampus and cortex (Figure 4B) which has been linked to beneficial neurophysiological changes, including neuronal remodeling and development, as well as neurotrophic responses to voluntary exercise [39]. Voluntary exercise has been demonstrated to exert beneficial neurological actions via increases in BDNF levels [40]. We identified several other transcriptomic changes that may be associated with AMI-mediated neuronal development, *e.g.* upregulation of non-imprinted gene in Prader-Willi Syndrome/Angelman syndrome 1 (*Nipa1*), Ena-vasodilator stimulated phosphoprotein (*Evl*) and sprouty protein with EVH-1 domain 1 (*Spred1*) [41]–[43]. With neurogenesis, increased metabolic support is required and we noted AMI-mediated regulation of transcripts controlling angiogenesis (brain-specific angiogenesis inhibitor 2, *Bai2*; receptor activity modifying protein 2, *Ramp2*), stress response (glutaredoxin 2, *Glrx2*; oxidation resistance 1, *Oxr1*) and energy regulation (peroxisome proliferative activated receptor gamma coactivator 1 beta, *Ppargc1b*; mitochondrial carrier homolog 2, *Mtch2*) [44]–[49].

Based on the data collected in this study, AMI appears to be an effective pro-cognitive agent in the old and cognitively-impaired 3×TgAD mice employed. It is highly likely that the therapeutic activity of AMI could be complex and mediated by the subtle modulation of multiple synergistic processes. Thus the therapeutic effect of AMI in aged 3×TgAD mice is likely to involve multiple systems including, alterations in amyloid processing, neurotrophin receptor modulation, neurogenesis, stress resistance and even anti-inflammatory actions. As AMI has demonstrated a significant beneficial action in the AD model used in this study the future re-assessment of well-tolerated, FDA-approved pharmacological agents, such as AMI, may yield novel, unexpected activities in different disease states. The divergence in cellular systems and pharmacological responses between ‘normal’ or ‘diseased’ states [10], [50] therefore may create pathology-specific drug effects that could usher in new waves of drug development based on studying approved drugs in novel situations.

## Supporting Information

Figure S1 **Assessment of beta and gamma secretase activity in 3×TgAD animals.** Effects of AMI treatment, compared to vehicle treatment, upon hippocampal or cortical beta- or gamma-secretase activity. Beta and gamma secretase activity is expressed as EDANS (5-((2-aminoethyl)amino)naphthalene-1-sulfonic acid) fluorescence per µg of specific tissue protein. Secretase activity in vehicle-treated 3×TgAD animals is indicated by black bars while secretase activity in AMI-treated 3×TgAD animals is indicated by grey bars. Hippocampal beta-secretase (A) and gamma-secretase (B) activity in vehicle- and AMI-treated 3×TgAD mice. Cortical beta-secretase (C) and gamma-secretase (D) activity in vehicle- AMI-treated 3×TgAD mice. Values in histograms represent mean ± SEM (n = 3).
(TIF)

Figure S2 **AMI-mediated alteration of synaptic factors and neuroprotective signaling in extended-culture primary hippocampal and cortical cells.** Murine hippocampal or cortical primary neurons were extracted as described in the Methods section. Cells were then allowed to mature for 21 days in culture before either, 3 additional days of culture with maintained 10 nM AMI stimulation (for PSD95 or synapsin I assessment) or acute stimulation with the same AMI dose for 20 minutes (for TrkB tyrosine phosphorylation and Akt-1 Ser-473 phosphorylation measurement). Representative western blots are represented with an associated histogram indicating quantifications of the western blot band intensities expressed as actin-normalized arbitrary absorbance units (AU). Values in each histogram represent mean ± SEM (n = 3). AMI effects on synapsin I (A) and PSD95 (B) expression in primary hippocampal cells after 21 days in culture. AMI effects on synapsin I (C) and PSD95 (D) expression in primary cortical cells after 21 days in culture. Acute AMI effects upon TrkB tyrosine phosphorylation (E) and Akt-1 Ser-473 phosphorylation (F) in hippocampal cells after 21 days in culture. Acute AMI effects upon TrkB tyrosine phosphorylation (G) and Akt-1 Ser-473 phosphorylation (H) in hippocampal cells after 21 days in culture.
(TIF)

Table S1 **Antisera employed for immunohistochemistry or western blot analysis.** For each antisera used the protein target, experimental dilution used and the proprietary source (with catalog number) is delineated.
(DOC)

Table S2 **AMI-mediated significant transcriptional alterations in adult 3×TgAD mouse hippocampus.** Z ratios of genes significantly up- (positive) or down-regulated (negative) in the hippocampus of 3×TgAD mice after AMI treatment compared to control vehicle treatment (AMI vs. vehicle (veh) z ratio).
(DOC)

Table S3 **AMI-mediated significant transcriptional alterations in adult 3×TgAD mouse cortex.** Z ratios of genes significantly up- (positive) or down-regulated (negative) in the cortex of 3×TgAD mice after AMI treatment compared to control vehicle treatment (AMI vs. vehicle (veh) z ratio).
(DOC)
