# Supplementary figures and images for: Amitriptyline-Mediated Cognitive Enhancement in Aged 3×Tg Alzheimer's Disease Mice Is Associated with Neurogenesis and Neurotrophic Activity
Source: PLoS One. 2011 Jun 27;6(6):e21660. doi: 10.1371/journal.pone.0021660 (PMC3124550; doi:10.1371/journal.pone.0021660)

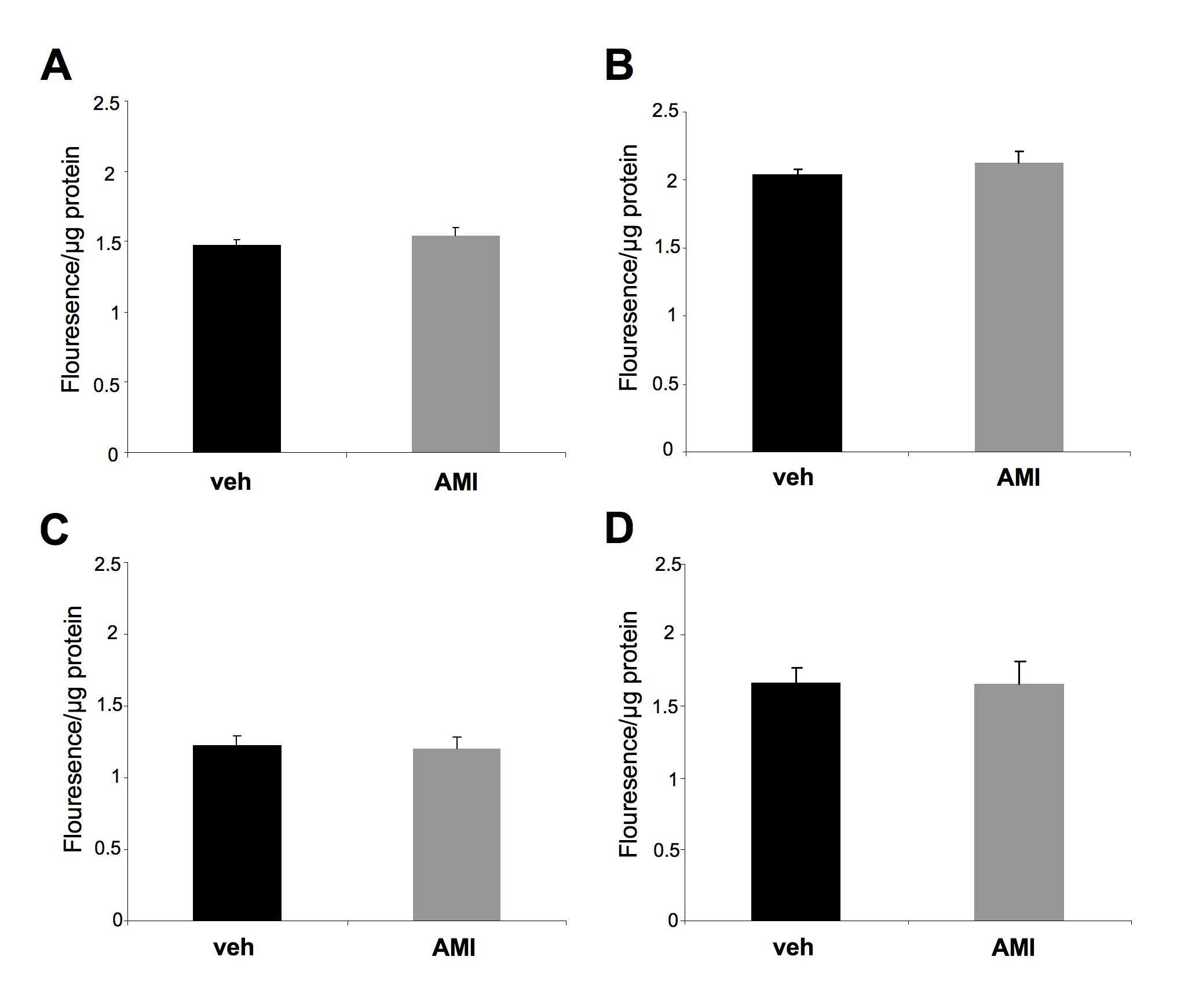

Supplement: Figure S1 — Assessment of beta and gamma secretase activity in 3×TgAD animals. Effects of AMI treatment, compared to vehicle treatment, upon hippocampal or cortical beta- or gamma-secretase activity. Beta and gamma secretase activity is expressed as EDANS (5-((2-aminoethyl)amino)naphthalene-1-sulfonic acid) fluorescence per µg of specific tissue protein. Secretase activity in vehicle-treated 3×TgAD animals is indicated by black bars while secretase activity in AMI-treated 3×TgAD animals is indicated by grey bars. Hippocampal beta-secretase (A) and gamma-secretase (B) activity in vehicle- and AMI-treated 3×TgAD mice. Cortical beta-secretase (C) and gamma-secretase (D) activity in vehicle- AMI-treated 3×TgAD mice. Values in histograms represent mean ± SEM (n = 3). (TIF) [file pone.0021660.s001.tif]

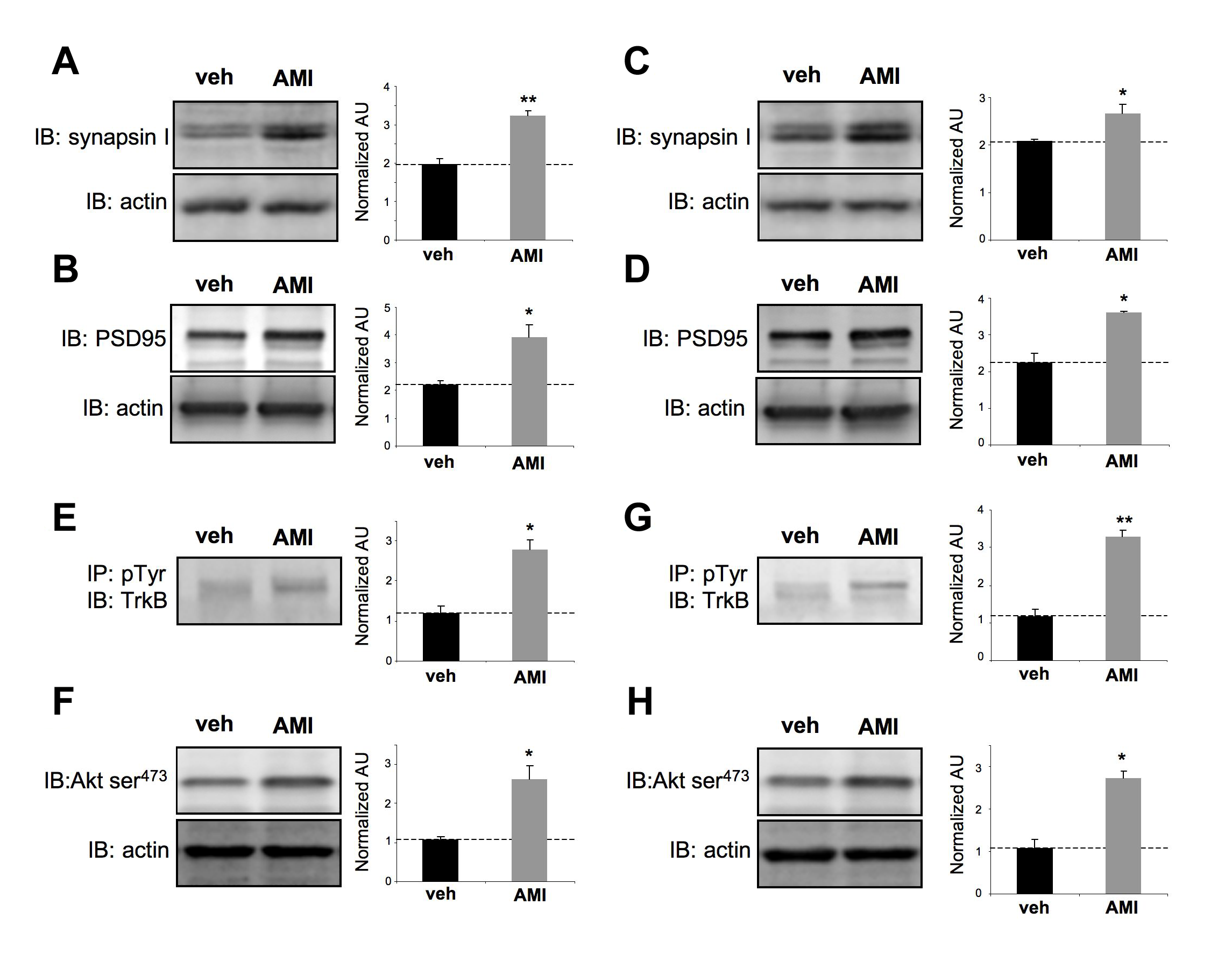

Supplement: Figure S2 — AMI-mediated alteration of synaptic factors and neuroprotective signaling in extended-culture primary hippocampal and cortical cells. Murine hippocampal or cortical primary neurons were extracted as described in the Methods section. Cells were then allowed to mature for 21 days in culture before either, 3 additional days of culture with maintained 10 nM AMI stimulation (for PSD95 or synapsin I assessment) or acute stimulation with the same AMI dose for 20 minutes (for TrkB tyrosine phosphorylation and Akt-1 Ser-473 phosphorylation measurement). Representative western blots are represented with an associated histogram indicating quantifications of the western blot band intensities expressed as actin-normalized arbitrary absorbance units (AU). Values in each histogram represent mean ± SEM (n = 3). AMI effects on synapsin I (A) and PSD95 (B) expression in primary hippocampal cells after 21 days in culture. AMI effects on synapsin I (C) and PSD95 (D) expression in primary cortical cells after 21 days in culture. Acute AMI effects upon TrkB tyrosine phosphorylation (E) and Akt-1 Ser-473 phosphorylation (F) in hippocampal cells after 21 days in culture. Acute AMI effects upon TrkB tyrosine phosphorylation (G) and Akt-1 Ser-473 phosphorylation (H) in hippocampal cells after 21 days in culture. (TIF) [file pone.0021660.s002.tif]
