# Supplementary material for: Amitriptyline-Mediated Cognitive Enhancement in Aged 3×Tg Alzheimer's Disease Mice Is Associated with Neurogenesis and Neurotrophic Activity
Source: PLoS One. 2011 Jun 27;6(6):e21660. doi: 10.1371/journal.pone.0021660 (PMC3124550; doi:10.1371/journal.pone.0021660)
Supplement: Table S1 — Antisera employed for immunohistochemistry or western blot analysis. For each antisera used the protein target, experimental dilution used and the proprietary source (with catalog number) is delineated. (DOC) [file pone.0021660.s003.doc]

**Table S1**. Antisera employed for immunohistochemistry or western blot analysis. For each antisera used the protein target, experimental dilution used and the proprietary source (with catalog number) is delineated.

| **Immunohistochemistry** |  |  |
| --- | --- | --- |
|  |  |  |
| *Protein Target* | *Experimental Dilution* | *Source* |
| Map2 | 1:1000 | Abcam - ab70218 |
| NeuN | 1:100 | Millipore - MAB377 |
| BrdU | 1:100 | Millipore - 05-633 |
| Amyloid β | 1:300 | Invitrogen - 71-5800 |
| AT8 | 1:1000 | Pierce - MN1020 |
|  |  |  |
| **Western Blot** |  |  |
|  |  |  |
| *Antisera* | *Experimental Dilution* | *Source* |
| synaptophysin | 1:6000 | Millipore - MAB5258 |
| synapsin I | 1:3000 | Sigma Aldrich - HPA000397 |
| PSD-95 | 1:5000 | Upstate - 05-494 |
| spinophilin | 1:2000 | Millipore - AB5669 |
| BDNF | 1:1000 | Santa Cruz Biotechnology - sc-8042 |
| NGF | 1:1000 | Abcam - ab63990 |
| TrkB | 1:1000 | Abcam - ab33655 |
| Ndrg4 | 1:1000 | Abcam - ab63990 |
| Flot2 | 1:1000 | Cell Signaling Technology - 3244 |
| Tau-AT180 | 1:1000 | Pierce-Thermo – MN1040 |
| Grlf1 | 1:1000 | Santa Cruz Biotechnology - sc-131536 |
| Neo1 | 1:1000 | Abcam - ab86577 |
| Rtn4 | 1:1000 | Lifespan Biosciences - LS-C19342 |
| Elavl2 | 1:1000 | Dr. Jack Keene, Duke University |
| Akt (phospho-Ser473) | 1:1000 | Cell Signaling Technology - 9271 |
| Actin | 1:5000 | Sigma Aldrich - A1978 |
| Amyloid β | 1:1000 | Sigma Aldrich - A8354 |
| agarose-conjugated phosphotyrosine | 1μg/100μg protein lysate | Santa Cruz Biotechnology - sc-508AC |
