# Supplementary material for: Amitriptyline-Mediated Cognitive Enhancement in Aged 3×Tg Alzheimer's Disease Mice Is Associated with Neurogenesis and Neurotrophic Activity
Source: PLoS One. 2011 Jun 27;6(6):e21660. doi: 10.1371/journal.pone.0021660 (PMC3124550; doi:10.1371/journal.pone.0021660)
Supplement: Table S2 — AMI-mediated significant transcriptional alterations in adult 3×TgAD mouse hippocampus. Z ratios of genes significantly up- (positive) or down-regulated (negative) in the hippocampus of 3×TgAD mice after AMI treatment compared to control vehicle treatment (AMI vs. vehicle (veh) z ratio). (DOC) [file pone.0021660.s004.doc]

**Table S2**. AMI-mediated significant transcriptional alterations in adult 3xTgAD mouse hippocampus. Z ratios of genes significantly up- (positive) or down-regulated (negative) in the hippocampus of 3xTgAD mice after AMI treatment compared to control vehicle treatment (AMI vs. vehicle (veh) z ratio).

| **ACCESSION** | **SYMBOL** | **GENE DEFINITION** | **AMI vs. veh (z ratio)** |
| --- | --- | --- | --- |
| NM_172739.4 | Grlf1 | Mus musculus glucocorticoid receptor DNA binding factor 1 | 5.137848225 |
| NM_031404.4 | Actl6b | Mus musculus actin-like 6B | 4.844884267 |
| NM_009568.2 | Zfp94 | Mus musculus zinc finger protein 94 | 4.164414985 |
| NM_133228.2 | Zfp87 | Mus musculus zinc finger protein 87 | 3.166236612 |
| NM_033524 | Spred1 | Mus musculus sprouty protein with EVH-1 domain 1, related sequence | 3.136712726 |
| NM_011192.3 | Psme3 | Mus musculus proteaseome (prosome, macropain) 28 subunit, 3 | 2.919598137 |
| NM_139236.3 | Nol6 | Mus musculus nucleolar protein family 6 (RNA-associated) | 2.904693755 |
| NM_178397.2 | Ubxd8 | Mus musculus UBX domain containing 8 | 2.897049601 |
| NM_029293.2 | Phpt1 | Mus musculus phosphohistidine phosphatase 1 | 2.833196284 |
| NM_133222.2 | Eltd1 | Mus musculus EGF, latrophilin seven transmembrane domain containing 1 | 2.789259682 |
| NM_133249.2 | Ppargc1b | Mus musculus peroxisome proliferative activated receptor, gamma, coactivator 1 beta | 2.75171807 |
| NM_028166.2 | 1600014C10Rik | Mus musculus RIKEN cDNA 1600014C10 gene | 2.740707894 |
| NM_008684.1 | Neo1 | Mus musculus neogenin 1 | 2.708901197 |
| NM_007616.3 | Cav1 | Mus musculus caveolin, caveolae protein 1 | 2.670364641 |
| NM_028130 | 2610020C11Rik | Mus musculus zinc finger protein 157 | 2.669841771 |
| NM_009517.2 | Zmat3 | Mus musculus zinc finger matrin type 3 | 2.662617351 |
| NM_011221.2 | Purb | Mus musculus purine rich element binding protein B | 2.602068894 |
| NM_019562.1 | Uchl5 | Mus musculus ubiquitin carboxyl-terminal esterase L5 | 2.600856712 |
| NM_178741.2 | Klhl8 | Mus musculus kelch-like 8 (Drosophila) | 2.549047551 |
| NM_020261.2 | Psg23 | Mus musculus pregnancy-specific glycoprotein 23 | 2.432408005 |
| NM_011400.2 | Slc2a1 | Mus musculus solute carrier family 2 (facilitated glucose transporter), member 1 | 2.432230385 |
| NM_019998.2 | Alg2 | Mus musculus asparagine-linked glycosylation 2 homolog (yeast, alpha-1,3-mannosyltransferase) | 2.409046748 |
| NM_183141.2 | Elfn2 | Mus musculus leucine rich repeat and fibronectin type III, extracellular 2 | 2.399008882 |
| NM_013470.1 | Anxa3 | Mus musculus annexin A3 | 2.339475067 |
| NM_183311.1 | B930076A02 | Mus musculus hypothetical proein B930076A02 | 2.338769197 |
| NM_019999.1 | Brp17 | Mus musculus paroxysmal nonkinesiogenic dyskinesia | 2.305066784 |
| NM_027872.3 | Slc46a3 | Mus musculus solute carrier family 46, member 3 | 2.272443481 |
| NM_009199.2 | Slc1a1 | Mus musculus solute carrier family 1 (neuronal/epithelial high affinity glutamate transporter, system Xag), member 1 | 2.26251829 |
| NM_013880.2 | Plcl2 | Mus musculus phospholipase C-like 2 | 2.249933073 |
| NM_145602.2 | Ndrg4 | Mus musculus N-myc downstream regulated gene 4 | 2.212441458 |
| NM_021344.2 | Tesc | Mus musculus tescalcin | 2.21122103 |
| NM_027481.2 | Sf4 | Mus musculus splicing factor 4 | 2.14703166 |
| NM_025822.3 | Rsrc1 | Mus musculus arginine/serine-rich coiled-coil 1 | 2.130769817 |
| NM_009067.3 | Ralbp1 | Mus musculus ralA binding protein 1 | 2.122204469 |
| NM_001008421.1 | Nol10 | Mus musculus nucleolar protein 10 | 2.102658654 |
| NM_180599.1 | Mfap3 | Mus musculus microfibrillar-associated protein 3, transcript variant 2 | 2.068343935 |
| NM_021420.2 | Stk4 | Mus musculus serine/threonine kinase 4 | 2.03776903 |
| NM_025948.2 | Lsm14a | Mus musculus LSM14 homolog A (SCD6, S. cerevisiae) | 2.03436744 |
| NM_025745.2 | 4933407N01Rik | Mus musculus endoplasmic reticulum lectin 1 | 2.020201235 |
| NM_145443.1 | L2hgdh | Mus musculus L-2-hydroxyglutarate dehydrogenase (L2hgdh), nuclear gene encoding mitochondrial protein | 2.005489812 |
| NM_010518.2 | Igfbp5 | Mus musculus insulin-like growth factor binding protein 5 | 1.975342051 |
| NM_009225.2 | Snrpb | Mus musculus small nuclear ribonucleoprotein B | 1.97015649 |
| NM_177253.1 | Cstf3 | Mus musculus cleavage stimulation factor, 3' pre-RNA, subunit 3, transcript variant 2 | 1.966351195 |
| NM_198600.1 | Pols | Mus musculus polymerase (DNA directed) sigma | 1.965174738 |
| NM_030121.3 | Asb8 | Mus musculus ankyrin repeat and SOCS box-containing protein 8 | 1.954493266 |
| NM_011729.1 | Ercc5 | Mus musculus excision repair cross-complementing rodent repair deficiency, complementation group 5 | 1.954415033 |
| NM_007944.2 | Eps15l1 | Mus musculus epidermal growth factor receptor pathway substrate 15-like 1 | 1.947303115 |
| NM_145217.2 | Diras1 | Mus musculus DIRAS family, GTP-binding RAS-like 1 | 1.894132079 |
| NM_023503.2 | Ing2 | Mus musculus inhibitor of growth family, member 2 | 1.884136 |
| NM_016920.1 | Atp6v0a1 | Mus musculus ATPase, H+ transporting, lysosomal V0 subunit A1 | 1.882752643 |
| NM_008748.2 | Dusp8 | Mus musculus dual specificity phosphatase 8 | 1.854925478 |
| NM_021299.1 | Ak3 | Mus musculus adenylate kinase 3 | 1.823169084 |
| NM_197990.2 | 1700025G04Rik | Mus musculus RIKEN cDNA 1700025G04 gene | 1.795957136 |
| NM_027352.2 | Gorasp2 | Mus musculus golgi reassembly stacking protein 2 | 1.795741488 |
| NM_008687.2 | Nfib | Mus musculus nuclear factor I/B | 1.783526213 |
| NM_027400.2 | Lman1 | Mus musculus lectin, mannose-binding, 1 | 1.775461186 |
| NM_010209.1 | Fh1 | Mus musculus fumarate hydratase 1 | 1.764107826 |
| NM_172635.2 | Patl1 | Mus musculus protein associated with topoisomerase II homolog 1 (yeast) | 1.76010169 |
| NM_027494.1 | Zcchc8 | Mus musculus zinc finger, CCHC domain containing 8 | 1.757741322 |
| NM_198614.2 | 4932409I22Rik | Mus musculus RIKEN cDNA 4932409I22 gene | 1.739652329 |
| NM_029662.1 | Mfsd2 | Mus musculus major facilitator superfamily domain containing 2 | 1.73601567 |
| NM_009320.3 | Slc6a6 | Mus musculus solute carrier family 6 (neurotransmitter transporter, taurine), member 6 | 1.730949 |
| NM_023322.2 | Zkscan14 | Mus musculus zinc finger with KRAB and SCAN domains 14 | 1.720466878 |
| NM_001038593.1 | Glrx2 | Mus musculus glutaredoxin 2 (thioltransferase), transcript variant 3 | 1.709911906 |
| NM_181391.2 | Chchd7 | Mus musculus coiled-coil-helix-coiled-coil-helix domain containing 7 | 1.692353806 |
| NM_133998.1 | 1810008A18Rik | Mus musculus RIKEN cDNA 1810008A18 gene | 1.690122956 |
| NM_025316.2 | Ndufb5 | Mus musculus NADH dehydrogenase (ubiquinone) 1 beta subcomplex 5, nuclear gene encoding mitochondrial protein | 1.678199209 |
| NM_145995.1 | 2700050L05Rik | Mus musculus RIKEN cDNA 2700050L05 gene, transcript variant 1 | 1.674628655 |
| NM_029879.2 | Rgs7bp | Mus musculus regulator of G-protein signalling 7 binding protein | 1.667029642 |
| NM_026418.2 | Rgs10 | Mus musculus regulator of G-protein signalling 10 | 1.660513484 |
| NM_145426.2 | Mfap3 | Mus musculus microfibrillar-associated protein 3, transcript variant 1 | 1.656257249 |
| NM_019637.3 | Styx | Mus musculus phosphoserine/threonine/tyrosine interaction protein | 1.63818016 |
| NM_022885.2 | Slc30a5 | Mus musculus solute carrier family 30 (zinc transporter), member 5 | 1.633244375 |
| NM_138314.2 | Nme7 | Mus musculus non-metastatic cells 7, protein expressed in (nucleoside-diphosphate kinase), transcript variant 1 | 1.627620737 |
| NM_001077707.1 | Shprh | Mus musculus SNF2 histone linker PHD RING helicase, transcript variant 1 | 1.62046452 |
| NM_007569.1 | Btg1 | Mus musculus B-cell translocation gene 1, anti-proliferative | 1.610561404 |
| NM_027901.2 | Gtf3c2 | Mus musculus general transcription factor IIIC, polypeptide 2, beta | 1.595769734 |
| NM_007965.2 | Evl | Mus musculus Ena-vasodilator stimulated phosphoprotein | 1.577611736 |
| NM_134149.1 | AI837181 | Mus musculus expressed sequence AI837181 | 1.573689068 |
| NM_201371.1 | Prmt8 | Mus musculus protein arginine N-methyltransferase 8 | 1.573277839 |
| NM_001040403.1 | Flot2 | Mus musculus flotillin 2, transcript variant 1 | 1.572586693 |
| NM_153584.1 | BC031353 | Mus musculus cDNA sequence BC031353 | 1.55725716 |
| NM_008950.1 | Psmc5 | Mus musculus protease (prosome, macropain) 26S subunit, ATPase 5 | 1.551240168 |
| NM_177472.4 | Btbd12 | Mus musculus BTB (POZ) domain containing 12 | 1.550947878 |
| NM_001081145.1 | Tigd2 | Mus musculus tigger transposable element derived 2 | 1.534653479 |
| NM_183034.1 | Plekhm1 | Mus musculus pleckstrin homology domain containing, family M (with RUN domain) member 1 | 1.522769942 |
| NM_010238.3 | Brd2 | Mus musculus bromodomain containing 2, transcript variant 1 | 1.51533575 |
| NM_011970.3 | Psmb2 | Mus musculus proteasome (prosome, macropain) subunit, beta type 2 | -1.507735515 |
| NM_198703.1 | Wnk1 | Mus musculus WNK lysine deficient protein kinase 1 | -1.508237287 |
| NM_009388.2 | Tkt | Mus musculus transketolase | -1.508962329 |
| NM_009096.2 | Rps6 | Mus musculus ribosomal protein S6 | -1.524407934 |
| NM_010580.1 | Itgb5 | Mus musculus integrin beta 5 | -1.52852312 |
| NM_144926.2 | Sez6l2 | Mus musculus seizure related 6 homolog like 2 | -1.540288992 |
| NM_025348.1 | Ndufa3 | Mus musculus NADH dehydrogenase (ubiquinone) 1 alpha subcomplex, 3 | -1.563302655 |
| NM_019634.2 | Tspan7 | Mus musculus tetraspanin 7 | -1.565588379 |
| NM_177618.4 | Wscd1 | Mus musculus WSC domain containing 1 | -1.566274246 |
| NM_001037740.1 | D330017J20Rik | Mus musculus RIKEN cDNA D330017J20 gene (D330017J20Rik), transcript variant 2 | -1.57694955 |
| NM_024227.2 | Mrpl28 | Mus musculus mitochondrial ribosomal protein L28, nuclear gene encoding mitochondrial protein | -1.607492242 |
| NM_016968.4 | Olig1 | Mus musculus oligodendrocyte transcription factor 1 | -1.611589788 |
| NM_020252.2 | Nrxn1 | Mus musculus neurexin I | -1.627425608 |
| NM_001037913.1 | LOC622404 | Mus musculus hypothetical protein LOC622404 | -1.635546347 |
| NM_012060.2 | Bcap31 | Mus musculus B-cell receptor-associated protein 31 | -1.642293438 |
| NM_138669.1 | Eif4a3 | Mus musculus eukaryotic translation initiation factor 4A, isoform 3 | -1.643517573 |
| NM_030693.1 | Atf5 | Mus musculus activating transcription factor 5 | -1.647470859 |
| NM_010684.2 | Lamp1 | Mus musculus lysosomal-associated membrane protein 1 | -1.659652767 |
| NM_021538.1 | Cope | Mus musculus coatomer protein complex, subunit epsilon | -1.66029184 |
| NM_029949.1 | Snapc3 | Mus musculus small nuclear RNA activating complex, polypeptide 3 | -1.676171926 |
| NM_001033263.1 | Centg1 | Mus musculus centaurin, gamma 1 | -1.710023586 |
| NM_177336.2 | B230373P09Rik | Mus musculus dynein, axonemal, heavy chain 1 | -1.71262795 |
| NM_011521.2 | Sdc4 | Mus musculus syndecan 4 | -1.719740893 |
| NM_178845.1 | Zfp277 | Mus musculus zinc finger protein 277, transcript variant 2 | -1.732964907 |
| NM_025605.2 | 2400001E08Rik | Mus musculus RIKEN cDNA 2400001E08 gene | -1.74010545 |
| NM_145550.2 | Yipf1 | Mus musculus Yip1 domain family, member 1 | -1.742532391 |
| NM_134130.1 | Abhd3 | Mus musculus abhydrolase domain containing 3 | -1.746678122 |
| NM_026330.2 | Nsmce1 | Mus musculus non-SMC element 1 homolog (S. cerevisiae) | -1.766090476 |
| NM_011306.3 | Rxrb | Mus musculus retinoid X receptor beta | -1.770435938 |
| NM_021494.1 | Rab6ip1 | Mus musculus Rab6 interacting protein 1 | -1.779657905 |
| NM_009194.2 | Slc12a2 | Mus musculus solute carrier family 12, member 2 | -1.781160214 |
| NM_020050.1 | Tmem9b | Mus musculus TMEM9 domain family, member B | -1.781542103 |
| NM_010591.1 | Jun | Mus musculus Jun oncogene | -1.805468955 |
| NM_011161.4 | Mapk11 | Mus musculus mitogen-activated protein kinase 11 | -1.870477846 |
| NM_001080385.1 | Clta | Mus musculus clathrin, light polypeptide (Lca), transcript variant 2 | -1.888086133 |
| NM_023716.1 | Tubb2b | Mus musculus tubulin, beta 2b | -1.89718271 |
| NM_010860.2 | Myl6 | Mus musculus myosin, light polypeptide 6, alkali, smooth muscle and non-muscle | -1.897437521 |
| NM_023716.1 | Tubb2b | Mus musculus tubulin, beta 2b | -1.915639708 |
| NM_133858.3 | 4930504E06Rik | Mus musculus RIKEN cDNA 4930504E06 gene | -2.004139909 |
| NM_134095.2 | D15Wsu75e | Mus musculus DNA segment, Chr 15, Wayne State University 75, expressed | -2.064287547 |
| NM_001081170.1 | Pacs2 | Mus musculus phosphofurin acidic cluster sorting protein 2 | -2.084377892 |
| NM_175286.3 | C430004E15Rik | Mus musculus RIKEN cDNA C430004E15 gene | -2.179969241 |
| NM_009254.2 | Serpinb6a | Mus musculus serine (or cysteine) peptidase inhibitor, clade B, member 6a | -2.240319757 |
| NM_013746.1 | Plekhb1 | Mus musculus pleckstrin homology domain containing, family B (evectins) member 1 | -2.393388451 |
| NM_172907.2 | Olfml1 | Mus musculus olfactomedin-like 1 | -2.46141323 |
| NM_029789.1 | Lass2 | Mus musculus LAG1 homolog, ceramide synthase 2 | -2.507248759 |
| NM_175229.3 | Srrm2 | Mus musculus serine/arginine repetitive matrix 2 | -2.592771678 |
| NM_029789.1 | Lass2 | Mus musculus LAG1 homolog, ceramide synthase 2 | -2.632966775 |
| NM_021356.2 | Gab1 | Mus musculus growth factor receptor bound protein 2-associated protein 1 | -2.654938455 |
| NM_029789.1 | Lass2 | Mus musculus LAG1 homolog, ceramide synthase 2 | -2.656686517 |
| NM_178111.3 | Trp53inp2 | Mus musculus transformation related protein 53 inducible nuclear protein 2 | -2.688883068 |
| NM_009155.3 | Sepp1 | Mus musculus selenoprotein P, plasma 1, transcript variant 1 | -2.754181879 |
| NM_008135.4 | Slc6a9 | Mus musculus solute carrier family 6 (neurotransmitter transporter, glycine), member 9 | -2.760658118 |
| NM_009940.2 | Coq7 | Mus musculus demethyl-Q 7 | -2.994640169 |
| NM_001038637.1 | Gng2 | Mus musculus guanine nucleotide binding protein (G protein), gamma 2, transcript variant 2 | -3.205355086 |
| NM_016969.1 | Myadm | Mus musculus myeloid-associated differentiation marker | -3.433346267 |
| NM_018870.2 | Pgam2 | Mus musculus phosphoglycerate mutase 2 | -3.50321724 |
| NM_024236.1 | Qdpr | Mus musculus quinoid dihydropteridine reductase | -3.789396972 |
| NM_009923.1 | Cnp | Mus musculus 2',3'-cyclic nucleotide 3' phosphodiesterase | -4.341644682 |
| NM_178774.3 | Prr18 | Mus musculus proline rich region 18 | -4.377184564 |
| NM_172476.4 | Tmc7 | Mus musculus transmembrane channel-like gene family 7 | -4.411672665 |
| XM_001479138.1 | LOC100047583 | PREDICTED: Mus musculus similar to apolipoprotein D | -4.493153492 |
| NM_153520.1 | Tmem10 | Mus musculus transmembrane protein 10 | -4.868448441 |
| NM_133977.2 | Trf | Mus musculus transferrin | -5.128979837 |
| NM_170669.2 | Rps15a | Mus musculus ribosomal protein S15a | -9.08751244 |
| NM_182995.1 | 6330503K22Rik | Mus musculus RIKEN cDNA 6330503K22 gene | -9.264442346 |
