# Supplementary material for: Amitriptyline-Mediated Cognitive Enhancement in Aged 3×Tg Alzheimer's Disease Mice Is Associated with Neurogenesis and Neurotrophic Activity
Source: PLoS One. 2011 Jun 27;6(6):e21660. doi: 10.1371/journal.pone.0021660 (PMC3124550; doi:10.1371/journal.pone.0021660)
Supplement: Table S3 — AMI-mediated significant transcriptional alterations in adult 3×TgAD mouse cortex. Z ratios of genes significantly up- (positive) or down-regulated (negative) in the cortex of 3×TgAD mice after AMI treatment compared to control vehicle treatment (AMI vs. vehicle (veh) z ratio). (DOC) [file pone.0021660.s005.doc]

**Table S3**. AMI-mediated significant transcriptional alterations in adult 3xTgAD mouse cortex. Z ratios of genes significantly up- (positive) or down-regulated (negative) in the cortex of 3xTgAD mice after AMI treatment compared to control vehicle treatment (AMI vs. vehicle (veh) z ratio).

| **ACCESSION** | **SYMBOL** | **GENE DEFINITION** | **AMI vs. veh (z ratio)** |
| --- | --- | --- | --- |
| NM_013470.1 | Anxa3 | Mus musculus annexin A3 | 5.140960945 |
| NM_194054.1 | Rtn4 | Mus musculus reticulon 4, transcript variant 1 | 4.442719265 |
| NM_011696.1 | Vdac3 | Mus musculus voltage-dependent anion channel 3 | 3.192751756 |
| NM_009221.2 | Snca | Mus musculus synuclein, alpha, transcript variant 2 | 3.053319272 |
| NM_020611.3 | Srd5a3 | Mus musculus steroid 5 alpha-reductase 3 | 3.045949769 |
| NM_130885.2 | Oxr1 | Mus musculus oxidation resistance 1 | 2.947765178 |
| NM_008786.1 | Pcmt1 | Mus musculus protein-L-isoaspartate (D-aspartate) O-methyltransferase 1 | 2.939708918 |
| NM_022992.2 | Arl6ip5 | Mus musculus ADP-ribosylation factor-like 6 interacting protein 5 | 2.910551938 |
| NM_025942.2 | Ola1 | Mus musculus Obg-like ATPase 1, transcript variant 1 | 2.84907851 |
| NM_027758.3 | Tbc1d9 | Mus musculus TBC1 domain family, member 9 | 2.819066212 |
| NM_172782.2 | Nxt2 | Mus musculus nuclear transport factor 2-like export factor 2 | 2.713376515 |
| NM_145358.1 | Camkk2 | Mus musculus calcium/calmodulin-dependent protein kinase kinase 2, beta | 2.669481327 |
| NM_001029978.1 | Tceal3 | Mus musculus transcription elongation factor A (SII)-like 3 | 2.605044179 |
| NM_175002.1 | BC025076 | Mus musculus membrane magnesium transporter 2 | 2.5949176 |
| NM_021535.3 | Smu1 | Mus musculus smu-1 suppressor of mec-8 and unc-52 homolog (C. elegans) | 2.592510471 |
| NM_011515.1 | Sybl1 | Mus musculus synaptobrevin like 1 | 2.568780099 |
| NM_207686.1 | Elavl2 | Mus musculus ELAV (embryonic lethal, abnormal vision, Drosophila)-like 2 (Hu antigen B) | 2.559919842 |
| NM_001081233.1 | EG433923 | Mus musculus predicted gene, EG433923 | 2.551241639 |
| NM_009460 | Sumo1 | Mus musculus SMT3 suppressor of mif two 3 homolog 1 (yeast) | 2.543556764 |
| NM_178726.3 | Ppm1l | Mus musculus protein phosphatase 1 (formerly 2C)-like | 2.494920585 |
| NM_029813.2 | 2210418O10Rik | Mus musculus RIKEN cDNA 2210418O10 gene, transcript variant 1, mRNA. | 2.466879658 |
| NM_153405.2 | Rbm45 | Mus musculus RNA binding motif protein 45 | 2.437380984 |
| NM_175245.2 | 2410129H14Rik | Mus musculus RIKEN cDNA 2410129H14 gene | 2.413542671 |
| NM_013760.3 | Dnajb9 | Mus musculus DnaJ (Hsp40) homolog, subfamily B, member 9 | 2.392359299 |
| NM_023429.3 | Ociad1 | Mus musculus OCIA domain containing 1 | 2.391334434 |
| NM_025887.2 | Rab5a | Mus musculus RAB5A, member RAS oncogene family | 2.390562277 |
| NM_146236.1 | Tceal1 | Mus musculus transcription elongation factor A (SII)-like 1 | 2.375978837 |
| NM_009807.2 | Casp1 | Mus musculus caspase 1 | 2.364496045 |
| NM_009460.2 | Sumo1 | Mus musculus SMT3 suppressor of mif two 3 homolog 1 (yeast) | 2.333090816 |
| NM_033080.2 | Nudt19 | Mus musculus nudix (nucleoside diphosphate linked moiety X)-type motif 19 | 2.320102804 |
| NM_007952.2 | Pdia3 | Mus musculus protein disulfide isomerase associated 3 | 2.301944313 |
| NM_026396.2 | Bxdc2 | Mus musculus brix domain containing 2 | 2.294989373 |
| NM_025289.1 | Tbrg1 | Mus musculus transforming growth factor beta regulated gene 1 | 2.276835328 |
| NM_019745.2 | Pdcd10 | Mus musculus programmed cell death 10 | 2.247987829 |
| NM_145358.1 | Camkk2 | Mus musculus calcium/calmodulin-dependent protein kinase kinase 2, beta | 2.239343555 |
| NM_028754.1 | 0610037L13Rik | Mus musculus RIKEN cDNA 0610037L13 gene | 2.226666092 |
| NM_053069.2 | Atg5 | Mus musculus autophagy-related 5 (yeast) | 2.171386605 |
| NM_009454.2 | Ube2e3 | Mus musculus ubiquitin-conjugating enzyme E2E 3, UBC4/5 homolog (yeast) | 2.167488151 |
| NM_025356.4 | Ube2d3 | Mus musculus ubiquitin-conjugating enzyme E2D 3 (UBC4/5 homolog, yeast) | 2.162981698 |
| XM_001477380.1 | LOC100047012 | Mus musculus similar to ubiquitin-conjugating enzyme UbcM2 | 2.137926336 |
| NM_178090.2 | Drbp1 | Mus musculus RNA binding motif protein 45 | 2.136067317 |
| NM_016753.4 | Lxn | Mus musculus latexin | 2.129831451 |
| NM_053102.2 | 15-Sep | Mus musculus selenoprotein | 2.113228219 |
| NM_016786.3 | Ube2k | Mus musculus ubiquitin-conjugating enzyme E2K (UBC1 homolog, yeast) | 2.103636125 |
| XR_032582.1 | LOC667609 | Mus musculus similar to mitochondrial ribosomal protein S5 | 2.071528881 |
| XM_001477065.1 | LOC100046918 | Mus musculus similar to Electron transferring flavoprotein, alpha polypeptide | 2.043379197 |
| NM_019758.2 | Mtch2 | Mus musculus mitochondrial carrier homolog 2 (C. elegans), nuclear gene encoding mitochondrial protein | 2.036501349 |
| NM_024186.1 | Ssbp2 | Mus musculus single-stranded DNA binding protein 2, transcript variant 1 | 1.989552589 |
| NM_198024.1 | Ranbp3l | Mus musculus RAN binding protein 3-like | 1.988171981 |
| NM_025706.2 | Tbc1d15 | Mus musculus TBC1 domain family, member 15 | 1.975366366 |
| NM_013663.4 | Sfrs3 | Mus musculus splicing factor, arginine/serine-rich 3 (SRp20) | 1.958451875 |
| XM_001474536.1 | LOC100045567 | Mus musculus similar to purine nucleoside phosphorylase | 1.95766364 |
| NM_020026.2 | B3galnt1 | Mus musculus UDP-GalNAc:betaGlcNAc beta 1,3-galactosaminyltransferase, polypeptide 1 | 1.942990464 |
| NM_008774.2 | Pabpc1 | Mus musculus poly A binding protein, cytoplasmic 1 | 1.936258774 |
| NM_024190.1 | Chmp1b | Mus musculus chromatin modifying protein 1B | 1.930543791 |
| NM_008465.4 | Kpna1 | Mus musculus karyopherin (importin) alpha 1 | 1.924513712 |
| NM_011406.1 | Slc8a1 | Mus musculus solute carrier family 8 (sodium/calcium exchanger), member 1 | 1.923889345 |
| NM_025432 | Trappc2 | Mus musculus trafficking protein particle complex 2 | 1.91465805 |
| NM_013876.3 | Rnf11 | Mus musculus ring finger protein 11 | 1.906866664 |
| NM_028719.1 | Cpne4 | Mus musculus copine IV | 1.902227735 |
| NM_019444.2 | Ramp2 | Mus musculus receptor (calcitonin) activity modifying protein 2 | 1.885598379 |
| NM_177730.3 | Impad1 | Mus musculus inositol monophosphatase domain containing 1 | 1.88039253 |
| NM_145609.1 | 8430410K20Rik | Mus musculus RIKEN cDNA 8430410K20 gene | 1.877270734 |
| NM_013659.3 | Sema4b | Mus musculus sema domain, immunoglobulin domain (Ig), transmembrane domain (TM) and short cytoplasmic domain, (semaphorin) 4B | 1.867100292 |
| NM_008073.2 | Gabrg2 | Mus musculus gamma-aminobutyric acid (GABA-A) receptor, subunit gamma 2, transcript variant 1 | 1.864788039 |
| NM_153578.2 | Nipa1 | Mus musculus non imprinted in Prader-Willi/Angelman syndrome 1 homolog (human) | 1.835253451 |
| NM_024186.1 | Ssbp2 | Mus musculus single-stranded DNA binding protein 2, transcript variant 1 | 1.822772169 |
| NM_026121.3 | Bag4 | Mus musculus BCL2-associated athanogene 4 | 1.812950939 |
| NM_024205.1 | 1200003C05Rik | Mus musculus RIKEN cDNA 1200003C05 gene | 1.810219273 |
| NM_009554.3 | Zfp37 | Mus musculus zinc finger protein 37 | 1.809619437 |
| NM_029394.3 | Snx24 | Mus musculus sorting nexing 24 | 1.790246162 |
| NM_027922.1 | Ankle2 | Mus musculus ankyrin repeat and LEM domain containing 2 | 1.77648994 |
| NM_026009.1 | 2610204L23Rik | Mus musculus coiled-coil domain containing 47 | 1.775166247 |
| NM_009744.3 | Bcl6 | Mus musculus B-cell leukemia/lymphoma 6 | 1.772778363 |
| NM_010199.2 | Fgf12 | Mus musculus fibroblast growth factor 12, transcript variant 2 | 1.76869606 |
| NM_145602.2 | Ndrg4 | Mus musculus N-myc downstream regulated gene 4 | 1.754438074 |
| NM_019580.4 | Gde1 | Mus musculus glycerophosphodiester phosphodiesterase 1 | 1.750688026 |
| NM_001098231.1 | Ppm2c | Mus musculus protein phosphatase 2C, magnesium dependent, catalytic subunit, nuclear gene encoding mitochondrial protein, transcript variant 1 | 1.746487083 |
| NM_146067.3 | C530044N13Rik | Mus musculus RIKEN cDNA C530044N13 gene | 1.745048259 |
| NM_013604.1 | Mtx1 | Mus musculus metaxin 1 | 1.741394873 |
| NM_133948.4 | Psip1 | Mus musculus PC4 and SFRS1 interacting protein 1 | 1.736817593 |
| NM_011068.1 | Pex11a | Mus musculus peroxisomal biogenesis factor 11a | 1.722379515 |
| NM_080563.3 | Rnf144a | Mus musculus ring finger protein 144A, transcript variant 2 | 1.712096283 |
| NM_011627.3 | Tpbg | Mus musculus trophoblast glycoprotein | 1.686124291 |
| NM_025443.2 | Pno1 | Mus musculus partner of NOB1 homolog (S. cerevisiae) | 1.682183031 |
| NM_001001804.1 | Abhd7 | Mus musculus abhydrolase domain containing 7 | 1.680914866 |
| NM_009796.2 | Capn7 | Mus musculus calpain 7 | 1.652950492 |
| NM_022885.2 | Slc30a5 | Mus musculus solute carrier family 30 (zinc transporter), member 5 | 1.648869704 |
| NM_008316.2 | Hus1 | Mus musculus Hus1 homolog (S. pombe) | 1.633602398 |
| NM_018768.2 | Stx8 | Mus musculus syntaxin 8 | 1.622925316 |
| NM_026273.1 | 4930453N24Rik | Mus musculus RIKEN cDNA 4930453N24 gene | 1.620066978 |
| NM_026425.1 | Nat5 | Mus musculus N-acetyltransferase 5 (ARD1 homolog, S. cerevisiae) | 1.617363534 |
| NM_010733.2 | Lrrn3 | Mus musculus leucine rich repeat protein 3, neuronal | 1.60360812 |
| NM_025272.2 | Atp6v0e | Mus musculus ATPase, H+ transporting, lysosomal V0 subunit E | 1.598745251 |
| NM_146236.1 | Tceal1 | Mus musculus transcription elongation factor A (SII)-like 1 | 1.597702846 |
| NM_172149.4 | Bnip1 | Mus musculus BCL2/adenovirus E1B interacting protein 1, NIP1 | 1.592507158 |
| NM_023697.1 | Rdh14 | Mus musculus retinol dehydrogenase 14 (all-trans and 9-cis) | 1.591181255 |
| NM_175194.2 | Slc25a16 | Mus musculus solute carrier family 25 (mitochondrial carrier, Graves disease autoantigen), member 16 , nuclear gene encoding mitochondrial protein | 1.583074244 |
| NM_025969.3 | 1700034H14Rik | Mus musculus RIKEN cDNA 1700034H14 gene | 1.580292274 |
| NM_016750.1 | H2afz | Mus musculus H2A histone family, member Z | 1.577209722 |
| NM_023697.1 | Rdh14 | Mus musculus retinol dehydrogenase 14 (all-trans and 9-cis) | 1.567783562 |
| NM_026164.2 | Pnpla8 | Mus musculus patatin-like phospholipase domain containing 8 | 1.566805611 |
| NM_016716.4 | Cul3 | Mus musculus cullin 3 | 1.550893404 |
| NM_177751.2 | Cnksr2 | Mus musculus connector enhancer of kinase suppressor of Ras 2 | 1.540815325 |
| NM_145393.2 | Ythdf2 | Mus musculus YTH domain family 2 | 1.529444652 |
| NM_028850.3 | Chic2 | Mus musculus cysteine-rich hydrophobic domain 2 | 1.513229404 |
| NM_027002.3 | Polr2d | Mus musculus polymerase (RNA) II (DNA directed) polypeptide D, transcript variant 1 | 1.507317783 |
| NM_016698.1 | Rnf10 | Mus musculus ring finger protein 10 | -1.500302091 |
| NM_001081241.2 | 2310066E14Rik | Mus musculus RIKEN cDNA 2310066E14 gene | -1.501678508 |
| NM_009006.2 | Map4k2 | Mus musculus mitogen-activated protein kinase kinase kinase kinase 2 | -1.501953379 |
| NM_028816.2 | Xpo6 | Mus musculus exportin 6 | -1.504665529 |
| NM_001001326.1 | St5 | Mus musculus suppression of tumorigenicity 5, transcript variant 1 | -1.520692042 |
| NM_025407.2 | Uqcrc1 | Mus musculus ubiquinol-cytochrome c reductase core protein 1 | -1.523775747 |
| NM_023126.2 | Rab8a | Mus musculus RAB8A, member RAS oncogene family | -1.526620997 |
| NM_178652.2 | Supt3h | Mus musculus suppressor of Ty 3 homolog (S. cerevisiae) | -1.528264221 |
| NM_198160.1 | Smarcc2 | Mus musculus SWI/SNF related, matrix associated, actin dependent regulator of chromatin, subfamily c, member 2 | -1.535201776 |
| NM_030180.2 | Usp54 | Mus musculus ubiquitin specific peptidase 54 | -1.541950971 |
| NM_029097.1 | Atp13a2 | Mus musculus ATPase type 13A2 | -1.561855797 |
| NM_177379.2 | Grit | Mus musculus Rho GTPase-activating protein | -1.563713363 |
| NM_010091.2 | Dvl1 | Mus musculus dishevelled, dsh homolog 1 (Drosophila) | -1.572871576 |
| NM_001025387.2 | Brd2 | Mus musculus bromodomain containing 2, transcript variant 2 | -1.581690833 |
| NM_028027.2 | D10Ertd610e | Mus musculus DNA segment, Chr 10, ERATO Doi 610, expressed | -1.583017593 |
| NM_016920.1 | Atp6v0a1 | Mus musculus ATPase, H+ transporting, lysosomal V0 subunit A1 | -1.590704581 |
| NM_011844.3 | Mgll | Mus musculus monoglyceride lipase | -1.593144923 |
| NM_144919.2 | Hdac11 | Mus musculus histone deacetylase 11 | -1.599991259 |
| NM_145533.1 | Smox | Mus musculus spermine oxidase | -1.610574375 |
| NM_001009948.1 | Nrsn2 | Mus musculus neurensin 2 | -1.618049978 |
| NM_008729.2 | Ctnnd2 | Mus musculus catenin (cadherin associated protein), delta 2 | -1.625241036 |
| NM_011588.2 | Trim28 | Mus musculus tripartite motif protein 28 | -1.634637296 |
| NM_029447.1 | Nln | Mus musculus neurolysin (metallopeptidase M3 family) | -1.642026791 |
| NM_008064.2 | Gaa | Mus musculus glucosidase, alpha, acid | -1.64744292 |
| NM_133224.1 | Atp13a1 | Mus musculus ATPase type 13A1 | -1.65581168 |
| NM_172615.3 | 1700021K19Rik | Mus musculus RIKEN cDNA 1700021K19 gene | -1.673757942 |
| NM_008792.3 | Pcsk2 | Mus musculus proprotein convertase subtilisin/kexin type 2 | -1.675493691 |
| NM_010048.2 | Dgcr2 | Mus musculus DiGeorge syndrome critical region gene 2 | -1.690698771 |
| NM_033371.2 | Ppp1r16a | Mus musculus protein phosphatase 1, regulatory (inhibitor) subunit 16A | -1.700302326 |
| NM_198411.2 | 2610204M08Rik | Mus musculus RIKEN cDNA 2610204M08 gene | -1.70267531 |
| NM_019392.2 | Tyro3 | Mus musculus TYRO3 protein tyrosine kinase 3 | -1.70734489 |
| NM_133789.2 | Strn4 | Mus musculus striatin, calmodulin binding protein 4, transcript variant 1 | -1.719164037 |
| NM_025996.1 | Tomm34 | Mus musculus translocase of outer mitochondrial membrane 34 | -1.727237551 |
| NM_007460.1 | Ap3d1 | Mus musculus adaptor-related protein complex 3, delta 1 subunit | -1.728846783 |
| NM_001006664.2 | Epb4.1l1 | Mus musculus erythrocyte protein band 4.1-like 1, transcript variant 3 | -1.743135002 |
| NM_009059.2 | Rgl2 | Mus musculus ral guanine nucleotide dissociation stimulator-like 2 | -1.7563585 |
| NM_001008548.2 | Pde2a | Mus musculus phosphodiesterase 2A, cGMP-stimulated | -1.763931425 |
| NM_030719.3 | Gats | Mus musculus opposite strand transcription unit to Stag3 | -1.769105651 |
| XM_001472990.1 | LOC100048858 | Mus musculus similar to Ribosomal protein S6 kinase, polypeptide 2 | -1.770088363 |
| NM_010471.2 | Hpca | Mus musculus hippocalcin | -1.771767345 |
| NM_026799.2 | Rnasen | Mus musculus ribonuclease III, nuclear | -1.790876616 |
| NM_001039581.1 | Abca3 | Mus musculus ATP-binding cassette, sub-family A (ABC1), member 3, transcript variant 2 | -1.815786785 |
| NM_172596.1 | Sec24c | Mus musculus SEC24 related gene family, member C (S. cerevisiae) | -1.837651029 |
| NM_015805.2 | Atp9b | Mus musculus ATPase, class II, type 9B | -1.86581589 |
| NM_011986.2 | Ncdn | Mus musculus neurochondrin | -1.869177409 |
| NM_021492.3 | Ap3b2 | Mus musculus adaptor-related protein complex 3, beta 2 subunit | -1.875430969 |
| NM_009931.1 | Col4a1 | Mus musculus procollagen, type IV, alpha 1 | -1.876493021 |
| NM_199307.1 | Ece1 | Mus musculus endothelin converting enzyme 1 | -1.878761979 |
| NM_183016.1 | Cdc42bpb | Mus musculus Cdc42 binding protein kinase beta | -1.89028378 |
| NM_021287.1 | Spnb3 | Mus musculus spectrin beta 3 | -1.891444383 |
| NM_138650.1 | Dgkg | Mus musculus diacylglycerol kinase, gamma | -1.896910298 |
| XM_487678 | Epb4.1l1 | Mus musculus erythrocyte protein band 4.1-like 1 | -1.899962156 |
| NM_026447.2 | Ppm1m | Mus musculus protein phosphatase 1M, transcript variant 1 | -1.91987971 |
| NM_021415.3 | Cacna1h | Mus musculus calcium channel, voltage-dependent, T type, alpha 1H subunit | -1.926869561 |
| XM_001473941.1 | LOC100045542 | Mus musculus similar to FERMRhoGEF (Arhgef) and pleckstrin domain protein 1 | -1.927208215 |
| XM_001479098.1 | LOC100047659 | Mus musculus hypothetical protein LOC100047659 | -1.93215985 |
| NM_130891.1 | Grip1 | Mus musculus glutamate receptor interacting protein 1, transcript variant 2 | -1.948974853 |
| NM_054095.2 | Necab2 | Mus musculus N-terminal EF-hand calcium binding protein 2 | -1.957212369 |
| NM_138306.1 | Dgkz | Mus musculus diacylglycerol kinase zeta | -1.964829009 |
| XM_127132.3 | AW555464 | Mus musculus expressed sequence AW555464 | -1.968244946 |
| NM_001024458.1 | Add1 | Mus musculus adducin 1 (alpha), transcript variant 1 | -1.989364096 |
| NM_001039086.1 | Rapgef1 | Mus musculus Rap guanine nucleotide exchange factor (GEF) 1, transcript variant 2 | -1.995726987 |
| NM_080437.2 | Celsr3 | Mus musculus cadherin, EGF LAG seven-pass G-type receptor 3 (flamingo homolog, Drosophila) | -2.003601014 |
| NM_013785.2 | Ihpk1 | Mus musculus inositol hexaphosphate kinase 1 | -2.007004371 |
| NM_028601.2 | Zmiz2 | Mus musculus zinc finger, MIZ-type containing 2, transcript variant 1 | -2.019619119 |
| NM_026799.2 | Rnasen | Mus musculus ribonuclease III, nuclear | -2.021686868 |
| XM_001479832.1 | LOC100048105 | Mus musculus similar to Ubc protein, transcript variant 1 | -2.02954158 |
| NM_147151.1 | Ehmt2 | Mus musculus euchromatic histone lysine N-methyltransferase 2, transcript variant short | -2.052868258 |
| NM_009208.1 | Slc4a3 | Mus musculus solute carrier family 4 (anion exchanger), member 3 | -2.053734676 |
| NM_153057.3 | Nomo1 | Mus musculus nodal modulator 1 | -2.065347462 |
| NM_178760.3 | Gpr107 | Mus musculus G protein-coupled receptor 107 | -2.076803417 |
| NM_028722.1 | 4121402D02Rik | Mus musculus RIKEN cDNA 4121402D02 gene | -2.086043151 |
| NM_015731.3 | Atp9a | Mus musculus ATPase, class II, type 9A | -2.098586017 |
| NM_016795.3 | Srpk1 | Mus musculus serine/arginine-rich protein specific kinase 1 | -2.11042215 |
| NM_026748.1 | Ints1 | Mus musculus integrator complex subunit 1 | -2.114533005 |
| NM_138306.1 | Dgkz | Mus musculus diacylglycerol kinase zeta | -2.130740488 |
| NM_178398.2 | Wipi2 | Mus musculus WD repeat domain, phosphoinositide interacting 2 | -2.131927596 |
| NM_001039000.1 | Kif5a | Mus musculus kinesin family member 5A | -2.139737776 |
| NM_010630.2 | Kifc2 | Mus musculus kinesin family member C2 | -2.145454358 |
| NM_175641.1 | Ltbp4 | Mus musculus latent transforming growth factor beta binding protein 4 | -2.146242748 |
| NM_144926.2 | Sez6l2 | Mus musculus seizure related 6 homolog like 2 | -2.161578089 |
| NM_001077696.1 | Hdac5 | Mus musculus histone deacetylase 5 (Hdac5), transcript variant 1 | -2.169062953 |
| NM_011256.1 | Pitpnm2 | Mus musculus phosphatidylinositol transfer protein, membrane-associated 2 | -2.173188078 |
| XR_031422.1 | LOC100045005 | Mus musculus similar to Deltex3 | -2.173573226 |
| NM_026380.3 | Rgs8 | Mus musculus regulator of G-protein signaling 8 | -2.203171002 |
| NM_030238.2 | Dync1h1 | Mus musculus dynein cytoplasmic 1 heavy chain 1 | -2.205052082 |
| NM_023128.2 | Palm | Mus musculus paralemmin | -2.228336401 |
| NM_020605.2 | Jph3 | Mus musculus junctophilin 3 | -2.306345382 |
| NM_019419.1 | Arl6ip1 | Mus musculus ADP-ribosylation factor-like 6 interacting protein 1 | -2.329372436 |
| NM_172766.2 | Nfrkb | Mus musculus nuclear factor related to kappa B binding protein | -2.341540099 |
| NM_008800.1 | Pde1b | Mus musculus phosphodiesterase 1B, Ca2+-calmodulin dependent | -2.356290881 |
| NM_011212.3 | Ptpre | Mus musculus protein tyrosine phosphatase, receptor type, E | -2.405396514 |
| NM_009208.2 | Slc4a3 | Mus musculus solute carrier family 4 (anion exchanger), member 3 | -2.470942647 |
| NM_011104.1 | Prkce | Mus musculus protein kinase C, epsilon | -2.476139289 |
| NM_013680.3 | Syn1 | Mus musculus synapsin I | -2.499962658 |
| NM_001002272.2 | Tro | Mus musculus trophinin (Tro), transcript variant 1 | -2.500833491 |
| NM_201394.1 | Plec1 | Mus musculus plectin 1, transcript variant 11 | -2.504977157 |
| NM_173071.1 | Bai2 | Mus musculus brain-specific angiogenesis inhibitor 2 | -2.513235125 |
| NM_009451.3 | Tubb4 | Mus musculus tubulin, beta 4 | -2.515936151 |
| NM_009932.2 | Col4a2 | Mus musculus collagen, type IV, alpha 2 | -2.551120309 |
| NM_207225.1 | Hdac4 | Mus musculus histone deacetylase 4 | -2.564216773 |
| NM_001002272.2 | Tro | Mus musculus trophinin (Tro), transcript variant 1 | -2.56784412 |
| NM_029274.2 | Wbp7 | Mus musculus WW domain binding protein 7 | -2.5790709 |
| NM_027937.1 | Caskin1 | Mus musculus CASK interacting protein 1 | -2.626334941 |
| NM_026718.2 | Ankrd13a | Mus musculus ankyrin repeat domain 13a | -2.628238147 |
| NM_175274.3 | Ttyh3 | Mus musculus tweety homolog 3 (Drosophila) | -2.660870774 |
| NM_001004156.2 | Plekhg5 | Mus musculus pleckstrin homology domain containing, family G (with RhoGef domain) member 5 | -2.667487424 |
| NM_029792.1 | B3gat1 | Mus musculus beta-1,3-glucuronyltransferase 1 (glucuronosyltransferase P) | -2.677721218 |
| NM_053262.3 | Hsd17b11 | Mus musculus hydroxysteroid (17-beta) dehydrogenase 11 | -2.770572509 |
| NM_009358.2 | Ppp2r5d | Mus musculus protein phosphatase 2, regulatory subunit B (B56), delta isoform | -2.79163149 |
| NM_001031814.1 | 2610207I05Rik | Mus musculus RIKEN cDNA 2610207I05 gene | -2.812636696 |
| NM_172449.1 | Bzrap1 | Mus musculus benzodiazapine receptor associated protein 1 | -2.816389092 |
| NM_172476.4 | Tmc7 | Mus musculus transmembrane channel-like gene family 7 | -2.90182463 |
| NM_019813 | Dbn1 | Mus musculus drebrin 1 | -2.967699361 |
| NM_029792.1 | B3gat1 | Mus musculus beta-1,3-glucuronyltransferase 1 (glucuronosyltransferase P) | -3.058795551 |
| NM_133788.1 | Icmt | Mus musculus isoprenylcysteine carboxyl methyltransferase | -3.084502379 |
| NM_021608.2 | Dctn5 | Mus musculus dynactin 5 | -3.112504837 |
| NM_010319.3 | Gng7 | Mus musculus guanine nucleotide binding protein (G protein), gamma 7, transcript variant 2 | -3.144030369 |
| NM_053167.1 | Trim9 | Mus musculus tripartite motif protein 9 | -3.229876391 |
| NM_010601.2 | Kcnh3 | Mus musculus potassium voltage-gated channel, subfamily H (eag-related), member 3 | -3.348494023 |
| NM_011286.2 | Rph3a | Mus musculus rabphilin 3A | -3.557112245 |
| NM_175641.1 | Ltbp4 | Mus musculus latent transforming growth factor beta binding protein 4 | -3.702925532 |
| NM_029929.2 | Vps33a | Mus musculus vacuolar protein sorting 33A (yeast) | -3.734526156 |
| NM_009933.2 | Col6a1 | Mus musculus procollagen, type VI, alpha 1 | -3.820020148 |
| NM_011268.2 | Rgs9 | Mus musculus regulator of G-protein signaling 9 | -4.079662959 |
| NM_173421.1 | BC030476 | Mus musculus cDNA sequence BC030476 | -4.11796615 |
| NM_182995.1 | 6330503K22Rik | Mus musculus RIKEN cDNA 6330503K22 gene | -6.964234388 |
| NM_170669.2 | Rps15a | Mus musculus ribosomal protein S15a | -10.25308589 |
